# The brief mind wandering three-factor scale (BMW-3)

**DOI:** 10.3758/s13428-024-02500-6

**Published:** 2024-09-11

**Authors:** Anna-Lena Schubert, Gidon T. Frischkorn, Kathrin Sadus, Matthew S. Welhaf, Michael J. Kane, Jan Rummel

**Affiliations:** 1 Department of Psychology, University of Mainz, Mainz, Germany; 2 Department of Psychology, University of Zurich, Zurich, Switzerland; 3 Department of Psychology, Heidelberg University, Heidelberg, Germany; 4 Department of Psychology, University of North Carolina at Greensboro, Greensboro, NC USA; 5 Department of Psychological and Brain Sciences, Washington University in St. Louis, St. Louis, MO USA

**Keywords:** Mind wandering, Task-unrelated thought, Questionnaire, Measurement

## Abstract

In recent years, researchers from different fields have become increasingly interested in measuring individual differences in mind wandering as a psychological trait. Although there are several questionnaires that allow for an assessment of people’s perceptions of their mind wandering experiences, they either define mind wandering in a very broad sense or do not sufficiently separate different aspects of mind wandering. Here, we introduce the Brief Mind Wandering Three-Factor Scale (BMW-3), a 12-item questionnaire available in German and English. The BMW-3 conceptualizes mind wandering as task-unrelated thought and measures three dimensions of mind wandering: unintentional mind wandering, intentional mind wandering, and meta-awareness of mind wandering. Based on results from 1038 participants (823 German speakers, 215 English speakers), we found support for the proposed three-factorial structure of mind wandering and for scalar measurement invariance of the German and English versions. All subscales showed good internal consistencies and moderate to high test–retest correlations and thus provide an effective assessment of individual differences in mind wandering. Moreover, the BMW-3 showed good convergent validity when compared to existing retrospective measures of mind wandering and mindfulness and was related to conscientiousness, emotional stability, and openness as well as self-reported attentional control. Lastly, it predicted the propensity for mind wandering inside and outside the lab (as assessed by in-the-moment experience sampling), the frequency of experiencing depressive symptoms, and the use of functional and dysfunctional emotion regulation strategies. All in all, the BMW-3 provides a brief, reliable, and valid assessment of mind wandering for basic and clinical research.

**Supplementary Information:**

The online version contains supplementary material available at 10.3758/s13428-024-02500-6.

Mind wandering describes the common experience of one’s thoughts drifting away from the here and now towards self-generated thoughts and feelings (Smallwood & Schooler, 2006). Adults spend, on average, 30% or more of their waking hours engaging in self-generated thoughts and feelings (Kane et al., 2007, 2017; Kawashima et al., 2023; Killingsworth & Gilbert, 2010; Schooler et al., 2011). On the one hand, such thoughts may facilitate prospective planning and yield relief from boredom during undemanding tasks (Baird et al., 2011; Mooneyham & Schooler, 2013; Steindorf & Rummel, 2017). On the other hand, mind wandering can negatively affect one’s general mood and one’s performance in tasks requiring high focus (Killingsworth & Gilbert, 2010; Mooneyham & Schooler, 2013; Randall et al., 2014; Rummel & Boywitt, 2014). Consequently, psychologists and neuroscientists from various fields have studied mind wandering extensively in recent years (Callard et al., 2013; Smallwood & Schooler, 2015).

The phenomenon of mind wandering is particularly relevant for clinical psychology, as it is implicated in psychopathologies such as clinical depression, obsessive–compulsive disorder (OCD), and attention deficit hyperactivity disorder (ADHD; Chaieb et al., 2022; Franklin et al., 2017; Seli et al., 2017). Individuals with these diagnoses experience specific thought states or contents more frequently than do those without these diagnoses. Individuals with clinical depression, for example, tend to ruminate. Rumination is a typical symptom of depression that can be regarded as a type of mind wandering (or off-task thinking) that is distinguished by its repetitive, negative content and its lack of control (J. M. Smith & Alloy, 2009; van Vugt et al., 2018). Similarly, individuals diagnosed with OCD experience obsessive–compulsive thoughts that can draw their attention away from an ongoing activity and thus interfere with performance or completion (Snyder et al., 2015). Some obsessive–compulsive thoughts can therefore also be regarded as an uncontrollable and unwanted type of mind wandering that is characterized by its persistent, intrusive, and distressing content (Seli et al., 2017). In comparison, individuals with ADHD (at least those with inattentive symptoms) are usually not absorbed in repetitive thoughts, but rather experience mental restlessness that can be characterized as excessive mind wandering, with multiple thoughts (at least some off-task) going on at the same time (Franklin et al., 2017; Mowlem et al., 2019).

In addition to being a core symptom of different psychopathologies, mind wandering and meta-awareness about one’s thought states also play a role in mindfulness-based cognitive therapy, which emphasizes attention to the present moment and nonjudgmental acceptance of one’s thoughts and feelings (Segal et al., 2018; van der Velden et al., 2015). Consequently, mindfulness-based interventions in general—and mindfulness-based cognitive therapy in particular—can reduce spontaneous mind wandering (Greenberg et al., 2018; Mrazek et al., 2013a, 2013b). Intriguingly, changes in mind wandering due to mindfulness-based cognitive therapy have even covaried with changes in self-reported depressive symptoms, which may suggest that mind wandering could play a role in the pathogenesis of clinical depression (Greenberg et al., 2018). Although these preliminary findings necessitate further research, they also suggest that psychotherapists could potentially use regular surveys of patients’ mind wandering to track changes in mental well-being.

To enable further research on intervention effects on mind wandering and individual differences in thought states, reliable and valid measurement tools are needed to assess the frequency, characteristics, and awareness of mind wandering episodes. Here we introduce the Brief Mind Wandering Three-Factor Scale (BMW-3), which has been developed to measure three dimensions of mind wandering: unintentional mind wandering, intentional mind wandering, and meta-awareness of mind wandering.

## Mind wandering as task-unrelated thought

In line with some previous conceptualizations, we define mind wandering as task-unrelated thoughts that occur while one is actively engaged in another ongoing mental activity (Seli et al., 2018; Smallwood & Schooler, 2006). While these thoughts can arise spontaneously and unintentionally, they may also be intentionally generated by redirecting attention away from the current activity (Carriere et al., 2013; Seli et al., 2016a, 2016b). Both types of mind wandering—unintentional and intentional—require attention to be diverted from an ongoing primary task, which distinguishes task-unrelated thoughts from stimulus-independent thoughts that may occur in the absence of any primary task (e.g., daydreaming; Mrazek, Phillips, et al., 2013a) or freely moving spontaneous thoughts that are characterized by wandering aimlessly from topic to topic (Christoff et al., 2016). Moreover, people can be more or less aware of their current thought focus, and they may differ in how quickly they become aware that their thoughts have wandered off (Smallwood et al., 2007). Hence, the meta-awareness of mind wandering is another important aspect of mind wandering that may affect its consequences or correlates (Schooler et al., 2011). Taken together, we identified three relevant dimensions of mind wandering: (1) unintentional off-task thought (during different kinds of activities), (2) intentional off-task thought (usually during mundane or unpleasant activities), and (3) awareness of task-unrelated thought (during different kinds of activities).

Although there are several reliable and validated scales measuring mind wandering and related constructs, none of the existing scales specifically captures all three dimensions of mind wandering defined above. Some questionnaires measure related constructs such as dispositional mindfulness (Brown & Ryan, 2003), the contents of thoughts occurring during resting state (i.e., stimulus-independent thought; Diaz et al., 2013), excessive mind wandering as a core symptom of ADHD (Mowlem et al., 2019), or imaginal processes more generally (Singer & Antrobus, 1963). However, these scales do not assess mind wandering phenomena in the narrower sense, as they have been investigated by researchers in laboratory and field studies.

Mind wandering in this narrower sense can be assessed with three recently developed questionnaires, the Mind Wandering Questionnaire (MWQ; Mrazek, Phillips, et al., 2013a), the Mind Wandering: Spontaneous and Deliberate scales (MW-S and MW-D; Carriere et al., 2013), and the Four Factors of Mind Wandering Questionnaire (4FMW; Lopez et al., 2023). The MWQ is a one-dimensional five-item scale that conceptualizes mind wandering as task-unrelated thoughts and is used to measure trait levels of unintentional (spontaneous) mind wandering. It has been validated across college, high school, and middle school samples, and has been found to have high internal consistency and to predict mind wandering assessed with ecological momentary assessment as well as the occurrence of task-unrelated thoughts during reading (Mrazek, Phillips, et al., 2013a; Ostojic-Aitkens et al., 2019). Because the MWQ only measures unintentional task-unrelated thoughts, however, it is not suitable for assessing individual differences in intentional mind wandering or mind-wandering meta-awareness and thus only sheds light on one facet of the broader construct.

Assessing mind wandering as a two-dimensional construct, the MW-S and MW-D scales measure unintentional (spontaneous) and intentional (deliberate) mind wandering with four items each. Validation of these scales in general population and college samples indicated good internal consistencies and moderate correlations between the two scales (0.29 to 0.50; Carriere et al., 2013). Furthermore, it has been demonstrated that spontaneous mind wandering, as assessed by the MW-S scale, is strongly associated with self-reported attentional control, mindfulness, cognitive and memory failure, and fidgeting (Carriere et al., 2013). Conversely, the correlations between these measures and deliberate mind wandering, as measured by the MW-D scale, were found to be only low to moderate, and only the MW-S but not the MW-D scale predicted mind wandering assessed with ecological momentary assessment as well as OCD and ADHD symptomatology (Carriere et al., 2013; Ostojic-Aitkens et al., 2019; Seli et al., 2015, 2017). This difference in the pattern of correlations demonstrates that the two scales measure different aspects of mind wandering, despite being moderately related to each other. Undoubtedly, this provides evidence for both the conceptual and empirical differentiation between unintentional and intentional mind wandering (but see concerns raised by Kane et al., 2021). However, both the MW-S and MW-D scales are unsuitable to measure mind wandering as task-related thought. This is because only one item in each scale arguably pertains to task-unrelated thoughts (MW-S: “I mind wander even when I'm supposed to be doing something else”; MW-D: “I find mind-wandering is a good way to cope with boredom”). Instead, the MW-S scale conceptualizes mind wandering more in line with the idea that mind wandering can be conceived of as a special case of spontaneous thought (Christoff et al., 2016). In addition, neither questionnaire allows for the assessment of meta-awareness of mind wandering as a separate dimension. Instead, only one item is incorporated in the MW-S scale that pertains to meta-cognitive states, specifically the feeling of lacking control over when one's mind wanders.

The 16-item 4FMW questionnaire offers the most comprehensive assessment of mind wandering (Lopez et al., 2023). Three of its subscales measure unintentional mind wandering as task-unrelated thought in (1) social situations (failure in social interaction scale), (2) prospective memory tasks (failure in interaction with objects), and (3) study-related tasks (inattention scale). The three subscales have been shown to be moderately to strongly correlated (0.35 to 0.78; Lopez et al., 2023), suggesting that they reflect a higher-order construct of unintentional mind wandering. Meanwhile, a fourth subscale of the 4FMW, not related to the other three, measures meta-awareness of mind wandering (unawareness scale). Validation of the 4FMW in student samples showed good internal consistencies of the four subscales and a high convergent validity of the total score with an Italian mind wandering questionnaire. Moreover, the 4FMW was able to distinguish between students with no or mild ADHD/OCD symptoms and students with moderate to extreme symptoms, supporting its usefulness for clinical research. However, the 4FMW only assesses unintentional but not intentional task-unrelated thoughts.

## Aims, development, and validation of the BMW-3

Considering the limitations of existing questionnaires, which each allow a reliable and valid assessment of mind wandering but either do not conceptualize mind wandering as task-unrelated thought (MW-S, MW-D) or do not include a measure of intentional mind wandering (MWQ, 4FMW), the current study aims to develop and validate a novel questionnaire, the BMW-3. The BMW-3 conceptualizes mind wandering as task-unrelated thought and measures three dimensions of mind wandering: unintentional mind wandering, intentional mind wandering, and meta-awareness of mind wandering. By capturing individual differences in both unintentional and intentional mind wandering, we expect the BMW-3 to reflect both the costs of mind wandering during demanding tasks and its benefits during undemanding tasks (Mooneyham & Schooler, 2013). In addition, the meta-awareness scale sheds a light on meta-cognitive states by assessing how quickly people become aware that their thoughts have wandered off.

In the present study, we validated the German and English versions of the BMW-3 across student and general population samples to evaluate the psychometric properties of the three subscales and their construct and criterion validity. Moreover, we conducted tests of measurement invariance across the German and English versions to determine whether data can be simply collapsed across different language versions or whether they need to be analyzed using structural equation models (SEMs) that account for differences in item intercepts or factor loadings between groups (Chen, 2008; Davidov et al., 2014).

With regard to the questionnaire’s convergent validity, we expected to find high correlations between the BMW-3 unintentional mind wandering scale and the MW-S scale as well as between the BMW-3 intentional mind wandering and the MW-D scales (Mrazek, Phillips, et al., 2013a). Moreover, we expected participants with a greater meta-awareness of mind wandering to also report greater dispositional mindfulness as measured with the MAAS (Michalak et al., 2011), as meta-awareness is crucial for various forms of mindfulness (Dunne et al., 2019).

To further validate the BMW-3, we related the three subscales to the Big Five personality traits, expecting to find different patterns of correlations for the three dimensions of mind wandering. We expected more conscientious people to experience fewer episodes of unintentional mind wandering and to also show a greater meta-awareness of thought states than less conscientious people. The reason for this is that meta-awareness necessitates meta-cognitive monitoring and control (Dunne et al., 2019), which are self-regulatory processes that rely on effective self-regulation, a trait that is associated with conscientiousness. However, previous research has only partially supported this hypothesis, with mixed evidence found regarding the relationship between conscientiousness and mind wandering (e.g., Caron et al., 2023; Jackson & Balota, 2012; Kane et al., 2017; Müller et al., 2021; Robison et al., 2020). Moreover, we expected neuroticism to predict episodes of unintentional mind wandering and to be negatively related to meta-awareness of thought states, as emotionally unstable individuals are known to worry a lot and experience problems with cognitive self-regulation, which is a prerequisite for meta-cognitive monitoring and control (Dunne et al., 2019; Klein & Robinson, 2019; Widiger & Oltmanns, 2017). Previous research indicated that thinking about personal concerns increases mind wandering (Klinger, 2013; Kopp et al., 2015; Robison et al., 2017), and, as such, a disposition to worry should be positively linked to episodes of unintentional mind wandering. Some evidence for the relationship between neuroticism and mind wandering comes from a study by Kane et al. (2017), who found a positive association between the two in the lab, but not in daily life. A recent study by Caron et al. (2023) supported the notion that more emotionally unstable individuals are more prone to both spontaneous and deliberate mind wandering, as measured by the MW-S and MW-D scales. However, the authors found only a weak and nonsignificant relationship between emotional stability and self-reported mind wandering during a sustained attention to response task. Overall, previous research on the relationship between neuroticism and mind wandering suggests that different situational factors (e.g., measurement inside vs. outside the lab, being in a situation inducing more or less emotional arousal, etc.) may affect the relationship between emotional stability and self-reported mind wandering. Lastly, we expected openness to predict episodes of intentional mind wandering, as people with high levels of this trait are typically more imaginative, tend to daydream, and enjoy flights of fantasy (Costa & McCrae, 2008; John et al., 2008; Sassenberg et al., 2023). While previous research on the correlation between mind wandering and openness may have been inconclusive, growing evidence suggests a link between openness and intentional mind wandering during nondemanding tasks (Ibaceta & Madrid, 2021; Robison et al., 2020; Rummel et al., 2022). We did not anticipate any relationships between the personality traits extraversion and agreeableness and the BMW-3, but tentative evidence suggests that they may be linked to mind wandering in certain circumstances (Caron et al., 2023; Pereira et al., 2020; Robison et al., 2020).

Moreover, we explored associations between the BMW-3 and different measures of cognition, such as general cognitive abilities, working memory capacity, and attentional control, because previous research has shown that individuals with better cognitive abilities tend to engage in less mind wandering, at least during challenging tasks (Ju & Lien, 2018; McVay & Kane, 2009; Randall et al., 2014; Voss et al., 2018). Furthermore, mind wandering has been shown to at least partially mediate the relationship between working memory capacity and higher-order cognitive performance such as reading comprehension (McVay & Kane, 2012; Unsworth & McMillan, 2013). Cognitive accounts of mind wandering propose that people with greater cognitive abilities are generally better able to maintain an on-task focus and are thus better able to resist distraction in terms of task-unrelated thoughts than people with worse cognitive abilities (Kane & McVay, 2012; Unsworth et al., 2021). Furthermore, the former are assumed to be better able to regulate their mind wandering behavior in accordance with current task demands than the latter (Kane et al., 2007; Robison et al., 2020; Rummel & Boywitt, 2014). Consequently, self-reported unintentional mind wandering can be expected to be negatively related with cognitive abilities and may be expected to be positively related with mind wandering meta-awareness, inasmuch as this awareness is central to redirecting attention back to the ongoing task after one’s thoughts have drifted off.

Lastly, we evaluated the criterion validity of the unintentional and intentional mind wandering scales by relating them to the momentary assessment of mind wandering experiences inside and outside the lab. In the lab, we used task-embedded thought probes that distinguished between task-related thoughts, unintentional mind wandering, and intentional mind wandering (Schubert et al., 2020; Seli et al., 2013; Weinstein et al., 2018). We anticipated the BMW-3 unintentional scale to predict rates of unintentional thought-probed mind wandering, and the BMW-3 intentional scale to predict rates of intentional thought-probed mind wandering. Outside the lab, we used an ecological momentary assessment (EMA) of mind wandering to see how well unintentional and intentional mind wandering assessed with the BMW-3 predicted mind wandering rates in daily life (Killingsworth & Gilbert, 2010; Smallwood & Schooler, 2015). Moreover, we assessed the clinical validity of the BMW-3 by studying its relationship with depressive symptoms, expecting that unintentional mind wandering would be particularly predictive of depression (Chaieb et al., 2022). In addition, we investigated whether the BMW-3 scales predicted the use of functional and dysfunctional emotion regulation strategies, as previous research has found a close link between mind wandering and mood, and even suggested that mind wandering can be leveraged to regulate negative emotions (Killingsworth & Gilbert, 2010; Kruger et al., 2020; Poerio et al., 2013). We therefore expected that the BMW-3 intentional mind wandering and meta-awareness scales might be related to the use of adaptive emotion regulation strategies, whereas the BMW-3 unintentional mind wandering scale might be related to the use of maladaptive emotion regulation strategies.

Taken together, our aims were (1) to develop a novel questionnaire that conceptualizes mind wandering as task-unrelated thought and measures three dimensions of mind wandering, the BMW-3, and (2) to validate German and English versions of the BMW-3 across student and general population samples, assess their psychometric properties, and evaluate their validity.

## Method

### Participants

To evaluate the psychometric properties and factor structure of the BMW-3, we collected data from 823 German-speaking participants between 18 and 65 years old (*M*_age_ = 24.94 years, *SD*_age_ = 8.14 years) in six different studies (see Table 1 for an overview) at Heidelberg University, the Swiss Federal Institute of Technology in Zürich, and the University of Mainz. In total, 589 female, 208 male, and seven nonbinary participants took part in the study (19 participants did not disclose their gender). Four study samples consisted of student participants, and two study samples consisted of participants sampled from the general population. Participants in all studies completed the BMW-3 in addition to other measures. We therefore used data from different studies to estimate the test–retest reliability and to evaluate different aspects of construct and criterion validity as described below. Moreover, 215 US-based English-speaking student participants from the University of North Carolina at Greensboro and the Greensboro Technical Community College were given the English version of the BMW-3 to test measurement invariance of the German and English versions. Informed consent was obtained from all individual participants included in the study.

**Table 1:**
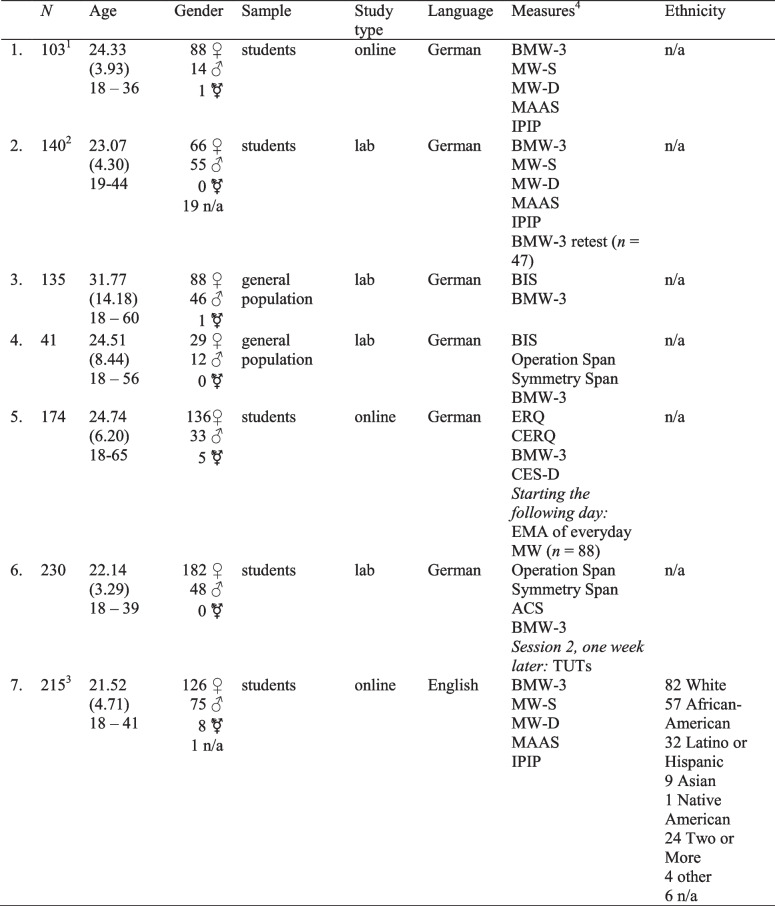
Overview of studies

MW-S: Mind-Wandering Spontaneous; MW-D: Mind-Wandering Deliberate; MAAS: Mindful Attention Awareness Scale; IPIP: International Personality Item Pool; BIS: Berlin Intelligence Structure Test; EMA: ecological momentary assessment; CES-D: Center of Epidemiological Research Depression Scale; ERQ: Emotion Regulation Questionnaire; CERQ: Cognitive Emotion-Regulation Questionnaire; TUTs: task-unrelated thoughts; ACS: Attentional Control Scale.^1^Three participants were excluded because they did not pass attention checks;^2^16 participants were excluded because they did not pass attention checks; ^3^ 64 participants were excluded because they did not pass attention checks (selecting a specific response option when instructed to do so). ^4^The order in which measures are listed reflects their task order in the respective studies

### Material

#### Brief Mind Wandering Three-Factor Scale (BMW-3)

The BMW-3 consists of three scales with four items each (see Table 2 for the English item translations and Table S1 for the original German items). For all items, the same five-point Likert response scale is used, with categories being labeled 0 = fully disagree, 1 = somewhat disagree, 2 = neutral, 3 = somewhat agree, and 4 = fully agree. Participants are instructed that they will see a couple of statements that generally describe where people may be with their thoughts in everyday life. They are asked to read each statement carefully and decide how much they agree or disagree with that statement. Moreover, they are told that there are no right or wrong answers, and they should just answer intuitively.

**Table 2:** Mean responses (*SD* in parentheses) and standardized factor loadings of all items

	Item	German version		English version	
		*M*	β	*M*	β
UI-MW 1	While listening to a presentation, my thoughts start to trail off unintentionally.	2.63 (1.01)	.67	3.01 (1.06)	.59
UI-MW 2	When watching TV, other things inadvertently cross my mind.	2.46 (1.11)	.53	2.94 (1.06)	.56
UI-MW 3	I am often absentminded.	2.16 (1.08)	.80	2.03 (1.18)	.64
UI-MW4	When I am engaged in an activity, my thoughts wander to other things all by themselves.	2.35 (1.08)	.76	2.49 (1.18)	.70
I-MW 1	I make my thoughts wander so that time passes faster.	1.99 (1.25)	.75	2.16 (1.37)	.88
I-MW 2	I deliberately allow my mind to wander to escape the daily grind.	1.79 (1.31)	.78	1.98 (1.38)	.43
I-MW 3	I distract myself in monotonous situations by letting my thoughts run free.	2.53 (1.19)	.75	2.40 (1.30)	.84
I-MW 4	I actively use the time during routine tasks to mull over other things in the meanwhile.	2.65 (1.20)	.32	2.45 (1.19)	.57
MA-MW 1^1^	It takes a very long time for me to notice that my thoughts have wandered off.	2.72 (1.04)	.81	2.49 (1.16)	.84
MA-MW 2	I quickly catch myself when I am not listening attentively.	2.97 (0.98)	.48	2.42 (1.09)	.87
MA-MW 3^1^	It takes me a while before I realize that I zoned out.	2.68 (1.03)	.85	2.41 (1.13)	.79
MA-MW 4	I immediately notice when my thoughts are not in the here and now.	2.51 (1.05)	.68	2.32 (1.17)	.47

UI-MW: unintentional mind wandering scale; I-MW: intentional mind wandering scale; MA = meta-awareness scale. *M* and β are used to represent the mean and standardized factor loading on the three factors, respectively, averaged across all studies. ^1^ Reverse-scored item (means and SDs were calculated after reverse scoring)

The Unintentional Mind Wandering (UI-MW) scale measures whether individuals’ thoughts wander off-task without their intention when they are engaged in other tasks or activities (e.g., “When I am engaged in an activity, my thoughts wander to other things all by themselves.”). The Intentional Mind Wandering (I-MW) scale measures whether people let their thoughts wander off-task intentionally while engaged in routine activities (e.g., “I deliberately allow my mind to wander to escape the daily grind.”). Higher UI-MW and I-MW scores reflect more unintentional and intentional mind wandering, respectively. The items of both scales are consistent with the conceptualization of mind wandering as task-unrelated thoughts. Moreover, they were formulated in a way that conveys that the mind wanders off unintentionally or intentionally in as simple terms as possible, to ensure that people from different educational and linguistic background understand the content in a similar manner.

Finally, the Meta-Awareness of Mind Wandering (MA-MW) scale measures how quickly an individual becomes aware that their mind has wandered off (“It takes a very long time for me to notice that my thoughts have wandered off.”). Items assess how long it takes to realize thoughts are not in the here and now, half referring to taking a long while and the other half to taking a short time. Hence, the former two should always be reverse-coded so that higher MA scores reflect greater meta-awareness of one’s thoughts.

#### Construct validity

##### **Mind Wandering: Spontaneous and Deliberate (MW-S and MW-D)**

We administered the four-item MW-S and MW-D scales (Carriere et al., 2013) in studies 1, 2, and 7 and used average scores to measure individual differences in spontaneous and deliberate mind wandering in everyday life. In studies 1 and 2, we used our own translation of the scales, and in study 7, we used the original English version of the scales.

##### **Mindful Attention Awareness Scale (MAAS)**

We administered the MAAS (Brown & Ryan, 2003) in studies 1, 2, and 7 and computed average scores of the 15 items to measure dispositional mindfulness. In studies 1 and 2, we used the German translation by Michalak et al. (2011), and in study 7, we used the original English version of the scale.

##### **International Personality Item Pool (IPIP)**

We administered the short 50-item version of the IPIP questionnaire (Goldberg, 1992) in studies 1, 2, and 7 to measure individual differences in the personality traits openness/intellect, conscientiousness, extraversion, agreeableness, and neuroticism (scored as emotional stability, with higher values indicating higher emotional stability) by computing average scores of the 10 items per scale. In studies 1 and 2, we used the German translation of the 50-item version by Ostendorf (n.d.), and in study 7, we used the original English 50-item version of the questionnaire.

##### **Berlin Intelligence Structure Test (BIS)**

We administered the short version of the BIS (Jäger et al., 1997), which consists of 15 subtests, in studies 3 and 4 to measure individual differences in in general cognitive abilities as reflected in the standardized component-specific scores for processing capacity, processing speed, memory, and creativity.

##### **Operation Span and Symmetry Span (O-Span und S-Span)**

In studies 4 and 5, we administered an operation and a symmetry span task from the German version of the complex span task battery (Rummel et al., 2019; Unsworth et al., 2005) to measure individual differences in working memory capacity. Participants’ performance was calculated as the number of to-be-stored items that were recalled in the correct position.

##### **Attentional Control Scale (ACS)**

In study 4, we administered the German version of the ACS as a 20-item measure of self-reported attentional control (Derryberry & Reed, 2002). The ACS consists of two subscales measuring attentional focusing, which is the ability to focus on an ongoing task and ignore distractors, and attentional shifting, which is the ability to shift to new tasks or between different tasks, with 9 and 11 items, respectively. For both scales, we computed average scores across the respective items.

#### Criterion validity

##### **Online thought-probing procedure**

We used the online thought-probing procedure (Schubert et al., 2020; Seli et al., 2013; Weinstein et al., 2018) to measure task-unrelated thoughts (TUT) in participants completing two levels of difficulty of an n-back task (Kirchner, 1958), a color-matching task (R. E. Smith & Bayen, 2004), and a memory-scanning task (Sternberg, 1969) in study 6. TUT rates were assessed at a separate measurement session than the BMW-3, usually seven days apart. At the beginning, participants learned that task-unrelated thoughts are thoughts unrelated to the current task and that experiencing such thoughts is a completely normal everyday phenomenon. Participants provided their thoughts after nine experimental trials with thought probes asking what they were thinking (“What have you been thinking about right now?”). Participants could choose between (1) thinking about the current task ("on-task"), (2) intentionally thinking about something different than the current task ("intentionally off-task"), or (3) unintentionally thinking about something different than the current task (“unintentionally off-task”). We calculated the number of intentional and unintentional off-task responses separately for the two conditions of each task. Separately for each variable, we removed any outlier values that were ± 3 SD from the sample mean, because some participants reported an unexpectedly high number of intentional TUTs in the more difficult conditions. On average, this led to the exclusion of 1.20% values. Participants reported to be intentionally off-task 6.39% (*SD* = 6.75%) of the time when probed, and to be unintentionally off-task 19.40% (*SD* = 9.25%) of the time when probed.

##### **Ecological momentary assessment (EMA) of mind wandering**

Participants’ mind wandering experiences were repeatedly assessed using momentary EMA methods with a smartphone application that ran on Android systems six times a day, with at least a 30-min break between notifications, for seven consecutive days. When the phone vibrated, participants were asked to say what they were thinking about and had 30 min to respond to the message. Options included the task they were currently working on, future tasks, personal worries and concerns, or something else. We also asked participants to type in a brief description of their thoughts and the task they were currently working on (data not reported here). When participants indicated they were not thinking about the current task, follow-up questions regarding the functionality of their off-task thoughts were asked (data not reported here). Mind wandering was measured as a dichotomous variable (coded as on-task if participants were thinking about the current task and off-task otherwise). On average, participants reported to be absentminded 33.60% (*SD* = 15.40%) of the times when probed throughout their day.

##### Depressive symptoms

 In study 5, participants completed the German version of the Center of Epidemiological Research Depression Scale (CES-D; Hautzinger et al., 2012; Radloff, 1977), which is a two-dimensional scale that measures the frequency with which one has experienced psychological and somatic depressive symptoms in the last seven days. In addition to using the CES-D score as a continuous variable, we also grouped participants into one group with a lower prevalence of depressive symptoms (CES-D < 22; *n* = 113) and another group with a higher prevalence of depressive symptoms (CES-D ≥ 22; *n* = 61), as suggested by the test manual.

##### **Emotion regulation**

In study 5, we administered the German versions of the 10-item Emotion Regulation Questionnaire and of the four-item rumination scale of the Cognitive Emotion-Regulation Questionnaire (CERQ; Garnefski & Kraaij, 2007; Loch et al., 2011) to assess participants’ tendency to regulate emotions using cognitive reappraisal, suppression, and rumination.

### Data analysis

We calculated descriptive statistics, item–total correlations, and internal consistencies using the R package *psych* (Revelle, 2020) and estimated structural equation models using the R package *lavaan* (Rosseel, 2012).

#### Structural equation models

**Fig. 1:**
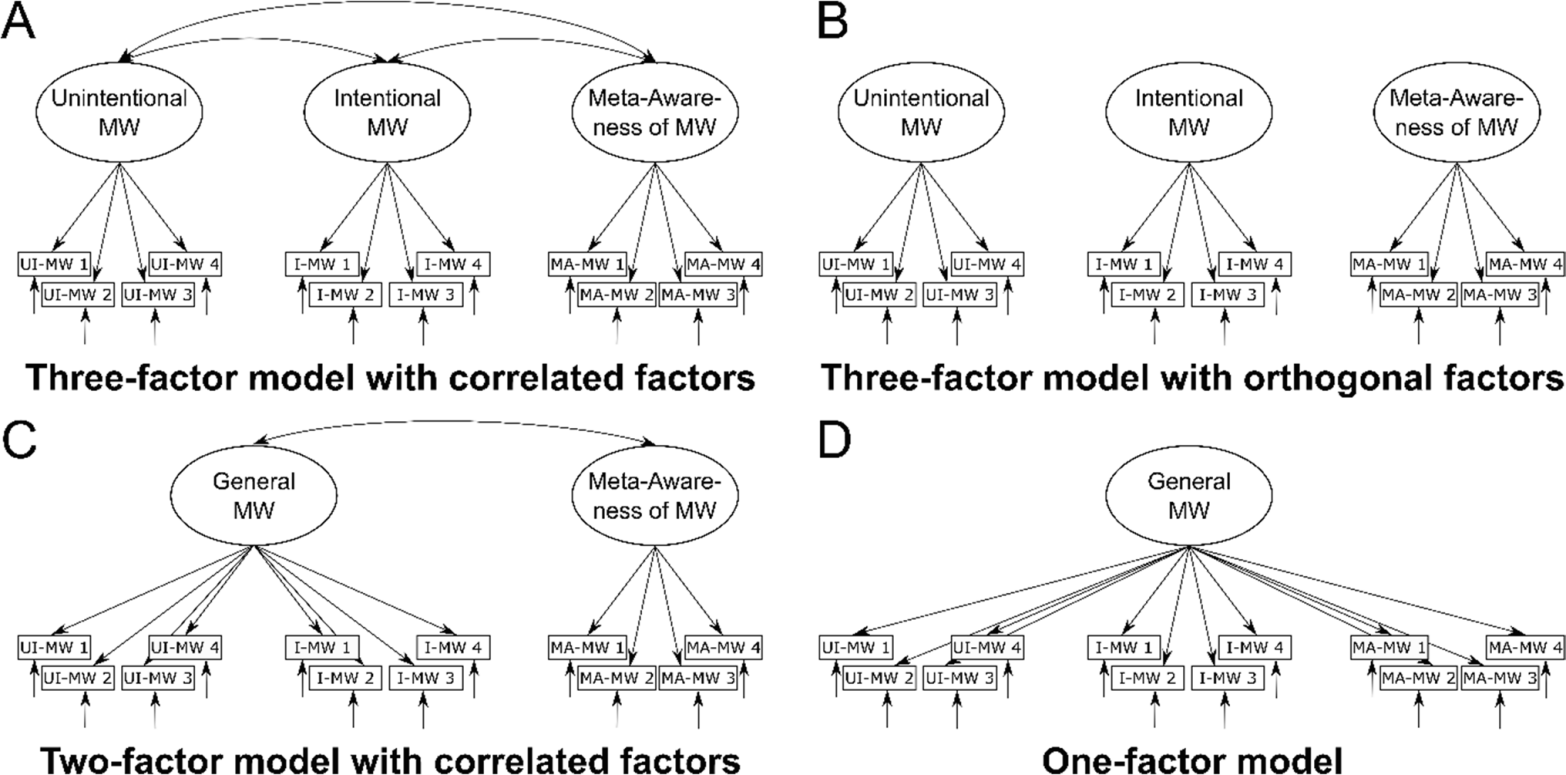
Graphical illustration of all compared CFA models. MW: mind wandering; UI-MW: unintentional mind wandering; I-MW: intentional mind wandering; MA-MW: meta-awareness of mind wandering

To evaluate the factorial validity of the BMW-3, we conducted a set of confirmatory factor analyses (see Fig. 1 for an overview of all compared models). We first specified and tested a three-factor model that consisted of a latent *unintentional mind wandering*, a latent *intentional mind wandering*, and a latent *meta-awareness* factor that were allowed to correlate freely with each other (see Fig. 1A). In addition, we also specified other models to ensure that the proposed factor structure provided the best account of the covariance structure. For this purpose, we specified a three-factor model with orthogonal latent factors (see Fig. 1B), a two-factor model with a latent *general mind wandering factor* loading onto the items of the unintentional and intentional mind wandering scales, in addition to a latent *meta-awareness* factor (see Fig. 1C), and a one-factor model with a latent *general mind wandering factor* (see Fig. 1D). Tests of measurement invariance of the German and English versions were conducted using the best-fitting model.

We assessed the convergent validity of the BMW-3 with related measures of mind wandering and mindfulness by computing correlations between the latent factors of the best-fitting BMW-3 model and those of the MW-S, MW-D, and MAAS. Each of the MW-S and MW-D scales had a single latent factor that loaded onto its four items, while the MAAS model included one latent factor that loaded onto three parcels, as also reported by Matsunaga (2008). All latent factors were allowed to correlate with each other.

Moreover, we evaluated the convergent and discriminant validity of the BMW-3 by regressing the latent factors of its best-fitting model onto the correlated latent factors of Big Five personality traits measured by the IPIP questionnaire. Measurement models of openness/intellect, conscientiousness, extraversion, agreeableness, and emotional stability each consisted of one latent factor loading onto three parcels. In addition, we also estimated latent correlations between BMW-3 factors and participants’ general cognitive abilities, their working memory capacity, and their self-reported attentional control, which were each modeled as a unidimensional first-order factors.

Finally, we evaluated the criterion validity of the BMW-3 by regressing latent factors of lab-probed unintentional and intentional task-unrelated thoughts on the latent factors of the best-fitting model of BMW-3 items. The measurement models of both types of task-unrelated thoughts consisted of one latent factor, loading on the number of off-task responses for each of the two conditions of the three experimental tasks.

To test whether individual differences in mind wandering predicted depressive symptoms measured with the CES-D, we regressed a latent depressive symptoms factor onto the latent factors of the best-fitting model of BMW-3 items. The measurement model of depressive symptoms consisted of one latent factor that loaded onto four parcels, with two parcels representing psychological and two parcels representing somatic depressive symptoms. Because CES-D scores were heavily skewed, we used robust Huber–White standard errors and a scaled test statistic asymptotically equal to the Yuan–Bentler test statistic to account for the nonnormal distribution of depressive symptoms.

In addition, we tested whether individual differences in mind wandering predicted emotion regulation strategies assessed with the ERQ and CERQ by regressing correlated latent factors of the emotion regulation strategies reappraisal, suppression, and rumination onto the latent factors of the best-fitting model of BMW-3 items. Each emotion regulation strategy was represented by one latent factor loading onto all items of the respective scale.

All SEMs were estimated with the full-information maximum likelihood (FIML) algorithm. We *z*-standardized all manifest variables, allowing us to fix their intercepts to 0. We evaluated goodness of fit based on the comparative fit index (CFI; Bentler, 1990) and the root mean square error of approximation (RMSEA; Browne & Cudeck, 1992) and compared the model fit of any two models with the Akaike information criterion (AIC; Akaike, 1973). We considered CFI values > 0.90 and RMSEA values < 0.08 to indicate acceptable model fit and CFI values > 0.95 and RMSEA values < 0.06 to indicate good model fit, as recommended by Browne and Cudeck (1992) and Hu and Bentler (1999). AIC differences ≥ 10 were interpreted to indicate a substantial advantage in relative model fit in direct model comparisons (Burnham & Anderson, 2003). The statistical significance of model parameters was assessed with the two-sided critical ratio test.

#### Generalized linear and mixed models

To evaluate the criterion validity of the BMW-3, we estimated a generalized mixed model with a logit link function using the R package *lme4* (Bates et al., 2020) to evaluate whether participants’ everyday mind wandering assessed by EMA could be predicted by the intentional and unintentional mind wandering scales of the BMW-3. We used self-reported thought states (0 = on-task, 1 = off-task) as the dependent variable. To account for circadian effects on mind wandering, the model included a count variable of thought probes (ranging from 0 to 5 over the course of the day) as a level-1 predictor. Moreover, it included a random intercept for participants and a random slope for day nested within participants to account for heterogeneity across participants and days (the random intercept and slope were uncorrelated). Participants’ intentional and unintentional mind wandering scores were entered as level-2 predictors.

Moreover, we tested whether the BMW-3 scales could classify between participants with high and low levels of depressive symptoms using a logistic regression with a logit link function.

### Transparency and openness

The BMW-3 questionnaire, the data supporting the findings of the study, and the statistical analysis code needed to reproduce the analyses in the manuscript are available in the Open Science Framework repository at https://osf.io/mxn3v/. The study materials will be shared upon request with the exception of the Berlin Intelligence Structure test, which is commercially licensed. Neither the study nor the analyses were preregistered.

## Results

Additional results can be found in the supplementary material, available online at https://osf.io/mxn3v/files/osfstorage/6537bd9f87852d1195a597b2.

### Validation of the German version of the BMW-3

Combined across all the studies consisting of German-speaking participants, items showed mean values ranging from 1.79 to 2.97 and homogeneous standard deviations ranging from 0.98 to 1.31, indicating that the BMW-3 is able to describe individual differences in self-perceptions of mind wandering. Deviations of items and scales from normal distributions were negligible (see Figure S1 for the distributions of individual item scores, and Table S2 for the correlation matrix), all skew values were ≤|1.10|, and all kurtosis values were ≤|1.22|. Internal consistencies of all three scales were acceptable to good, with α = 0.78 for the UI-MW scale, α = 0.73 for the I-MW scale, and α = 0.80 for the MA-MW scale.

#### **Factor structure**

**Fig. 2:**
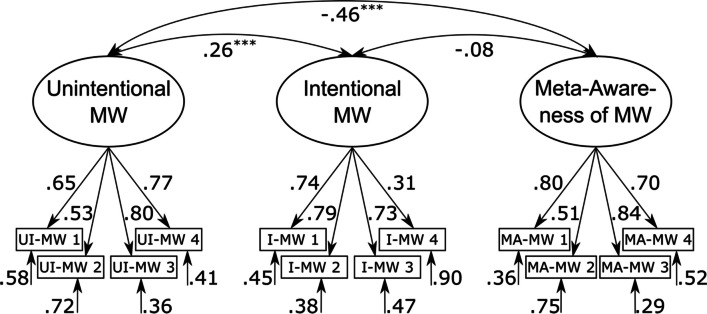
Graphical illustration of the best-fitting CFA model of the German version. Standardized factor loadings, correlation coefficients, and residual variances are shown next to paths. MW: mind wandering; UI-MW: unintentional mind wandering; I-MW: intentional mind wandering; MA-MW: meta-awareness of mind wandering. ^*^ *p* < .01. ^***^ *p* < .001*. N* = 823

The three-factor model consisting of a latent *unintentional mind wandering*, a latent *intentional mind wandering*, and a latent *meta-awareness* of mind wandering factor that were allowed to correlate freely with each other (see Fig. 2) provided the best account of the data, χ^2^(51) = 167.85, *p* < 0.001, CFI = 0.96, RMSEA = 0.05 (90% CI = [0.04; 0.06]). In addition, an alternative model with three orthogonal factors also provided an acceptable account of the data, χ^2^(54) = 336.86, *p* < 0.001, CFI = 0.91, RMSEA = 0.08 (90% CI = [0.07; 0.09]), but model fit was worse in comparison to the three-factor model with correlated factors, ΔAIC = 163. In comparison, both the alternative two-factor model, with a latent *general mind wandering factor* and a latent *meta-awareness of mind wandering* factor, χ^2^(53) = 914.75, *p* < 0.001, CFI = 0.72, RMSEA = 0.14 (90% CI = [0.13; 0.15]), ΔAIC = 743, and the alternative one-factor model, with a latent *general mind wandering factor*, χ^2^(54) = 1632.87, *p* < 0.001, CFI = 0.50, RMSEA = 0.19 (90% CI = [0.18; 0.20]), ΔAIC = 1,459, provided a poor account of the data.

Subsequently, we evaluated the psychometric properties of the BMW-3 based on the best-fitting three-factor solution with correlated factors (see Fig. 2). Factor loadings were satisfactory (all βs ≥ 0.50) except for the fourth item of the I-MW scale (β = 0.31). We nevertheless retained this item because it did not fall below the proposed cutoff value of 0.30 (Merenda, 2019). We observed a negative correlation between meta-awareness and unintentional mind wandering, *r* =  − 0.46, *p* < 0.001, 90% CI = [− 0.52; − 0.41], which indicates that individuals who were more aware of their thought states were also less likely to experience unwanted episodes of mind wandering. Most importantly, intentional and unintentional mind wandering were only weakly correlated, *r* = 0.26, *p* < 0.001, 90% CI = [0.20; 0.33], which indicates that the questionnaire can successfully distinguish between the two types of mind wandering.

#### **Stability**

We estimated the test–retest correlation of BMW-3 scales using a subsample of 47 participants from study 2 who completed the questionnaire a second time about half a year after the first assessment. All scales showed moderate to high test–retest correlations. The unintentional mind wandering scale showed the highest stability across the two measurement sessions, *r*_tt_ = 0.73, *p* < 0.001, 90% CI = [0.56; 0.84], while test–retest correlations of the intentional mind wandering scale, *r*_tt_ = 0.41, *p* = 0.004, 90% CI = [0.14; 0.62] and the meta-awareness of mind wandering scale, *r*_tt_ = 0.55, *p* < 0.001, 90% CI = [0.31; 0.72], were slightly lower (with wider CIs). The test–retest correlation for the meta-awareness scale did not differ significantly from either the correlation for the unintentional or the intentional scale, *p* > 0.125, whereas the test–retest correlation for the unintentional scale was significantly larger than that for the intentional scale, *p* = 0.019.

#### Construct validity

***Measures of mind wandering and mindfulness.*** We assessed the convergent validity of the BMW-3 with related measures of mind wandering and mindfulness by computing correlations between the latent factors of the three-factor BMW-3 model and those of the MW-S, MW-D, and MAAS in a subsample of 177 participants from studies 1 and 2 (see Table 3). The SEM provided a good account of the data, χ^2^(238) = 375.22, *p* < 0.001, CFI = 0.93, RMSEA = 0.06 (90% CI = [0.05; 0.07]).

**Table 3:** Means, standard deviations, and latent correlations between the BMW-3 factors (UI-MW, I-MW, MA-MW), the MW-S, MW-D, and MAAS

	*M*	*SD*	UI-MW	I-MW	MA-MW	MW-S	MW-D
UI-MW	2.50	0.80					
I-MW	2.19	0.88	.33^***^				
MA-MW	2.78	0.78	− .54^***^	− .15			
MW-S	4.32	1.43	.75^***^	.08	− .49^***^		
MW-D	4.68	1.27	.21^*^	.77^***^	− .08	.16	
MAAS	3.75	0.74	− .68^***^	− .19^*^	.45^***^	− .52^***^	− .11

UI-MW: unintentional mind wandering; I-MW: intentional mind wandering; MA-MW: meta-awareness of mind wandering; MW-S: Mind Wandering: Spontaneous; MW-D: Mind Wandering: Spontaneous; MAAS: Mindful Attention Awareness Scale. *M* and *SD* are used to represent the mean and standard deviation, respectively. *N* = 253

^*^ *p* < .05. ^***^ *p* < .001

As expected, we observed a high correlation between the UI-MW and MW-S scales, *r* = 0.75, *p* < 0.001, 90% CI = [0.68; 0.83], indicating that both scales measure unintentional/spontaneous mind wandering. The UI-MW and MW-D scales had a low correlation, showing a distinction between unintentional and deliberate mind wandering. Further, the moderate negative correlation between UI-MW and the MAAS suggests that mindful people are less prone to unwanted mind wandering.

We also observed a high correlation between the I-MW and MW-D scales, *r* = 0.77, *p* < 0.001, 90% CI = [0.69; 0.85], indicating that both scales measure intentional/deliberate mind wandering. Furthermore, the I-MW scale was not correlated with the MW-S scale and only weakly correlated with the MAAS, showing a clear distinction between intentional and spontaneous mind wandering as well as mindfulness.

We only observed a moderate correlation between the MA-MW scale and the MAAS, *r* = 0.45, *p* < 0.001, 90% CI = [0.33; 0.56], demonstrating that the two scales measure different facets of mindful awareness. Additionally, a moderately negative correlation between MA-MW and MW-S (in parallel to that between MA-MW and UI-MW) indicated that those individuals who become quickly aware of their mind wandering off tend to be less prone to spontaneous mind wandering. The MA-MW and the MW-D scales were not correlated (in parallel to MA-MW and I-MW), suggesting that they represent distinct factors.

***Big Five personality traits.*** We evaluated the convergent and discriminant validity of the BMW-3 by regressing the latent factors of the three-factor model onto the correlated latent factors of Big Five personality traits measured by the IPIP questionnaire in a subsample of 177 participants from studies 1 and 2. The SEM provided a good account of the data, χ^2^(324) = 439.43, *p* < 0.001, CFI = 0.95, RMSEA = 0.05 (90% CI = [0.03; 0.06]). The three BMW-3 factors showed both overlapping and distinct associations with Big Five personality traits (see Table 4). Those who scored higher on conscientiousness and emotional stability were less prone to unintentional mind wandering and quicker to become aware of their wandering mind, whereas those with higher openness to experience showed a greater likelihood to intentionally let their minds wander during routine activities. Notably, more conscientious people were less likely to let their minds wander than less conscientious individuals.

**Table 4:** Means and standard deviations of Big Five personality traits and results of the latent regression of BMW-3 factors on Big Five personality traits

			Dependent variable							
			UI-MW			I-MW			MA-MW	
	*M*	*SD*	β	90% CI	β		90% CI	β		90% CI
O	3.84	0.56	-.13	-.28 – .01	.21^*^		.05 – .37	.02		-.13 – .16
C	3.55	0.65	-.27^**^	-.41 – -.14	-.22^*^		-.37 – -.06	.33^***^		.20 – .47
E	3.14	0.82	.02	-.13 – .17	.05		-.13 – .22	.12		-.03 – .27
A	4.13	0.58	-.04	-.16 – .09	-.02		-.16 – .12	.01		-.11 – .14
N	2.94	0.81	-.39^***^	-.53 – -.26	-.01		-.17 – .15	.19^*^		.06 – .33

UI-MW: unintentional mind wandering; I-MW: intentional mind wandering; MA-MW: meta-awareness of mind wandering; O: openness to experience; C: conscientiousness; E: extraversion; A: agreeableness; N: emotional stability (i.e., higher values indicate higher emotional stability). *M* and *SD* are used to represent the mean and standard deviation, respectively. *N* = 253. ^*^ *p* < .05. ^**^ *p* < .01. ^***^ *p* < .001

***Cognitive abilities.*** To further investigate the convergent and discriminant validity of the BMW-3, we evaluated the relationship between BMW-3 factors and participants’ general cognitive abilities, their working memory capacity, and their self-reported attentional control (for descriptive statistics and zero-order correlations among these measures, see Tables S3–S5 in the supplementary material.).

In a subsample of 176 participants from studies 3 and 4 (see Table S3 for descriptives and zero-order correlations), we found no significant correlation between any BMW-3 factor and participants’ general cognitive abilities as measured with the BIS. The model showed an overall good model fit, χ^2^(114) = 166.89, *p* = 0.001, CFI = 0.92, RMSEA = 0.05 (90% CI = [0.03; 0.07]). More intelligent participants were equally prone to unintentional and intentional mind wandering as less intelligent participants, *r*_UN,g_ = 0.03, *p* = 0.802, 90% CI = [− 0.14; 0.19], and *r*_IN,g_ = 0.11, *p* = 0.262, 90% CI = [− 0.05; 0.27], respectively. In addition, participants’ cognitive ability was also unrelated to their meta-awareness of mind wandering, *r*_MA,g_ =  − 0.04, *p* = 0.647, 90% CI = [− 0.19; 0.11]. Fixing these correlations between BMW-3 factors and cognitive abilities to 0 did not affect model fit, ∆AIC = 4.50, which supports the interpretation that participants’ BMW-3 scores were unrelated to their cognitive abilities.

Next, we used a subsample of 271 participants from studies 4 and 6 (see Table S4 in the supplement for descriptives and zero-order correlations) to estimate the relationship between BMW-3 factors and working memory capacity. Again, we found no significant correlation between any BMW-3 factor and participants’ working memory capacity as measured with the operation span and symmetry span tasks. The model showed an overall good model fit, χ^2^(115) = 152.80, *p* = 0.011, CFI = 0.97, RMSEA = 0.04 (90% CI = [0.02; 0.05]). Participants with higher working memory capacity were equally prone to unintentional and intentional mind wandering as those with lower working memory capacity, *r*_UN,WMC_ =  − 0.05, *p* = 0.624, 90% CI = [− 0.23; 0.13], and *r*_IN,WMC_ =  − 0.05, *p* = 0.622, 90% CI = [− 0.22; 0.12], respectively. In addition, participants’ cognitive ability was also unrelated to their meta-awareness of mind wandering, *r* = 0.02, *p* = 0.880, 90% CI = [− 0.15; 0.18]. Fixing these correlations between BMW-3 factors and working memory capacity to 0 did not affect model fit, ∆AIC = 5.00, which supports the interpretation that participants’ BMW-3 scores were unrelated to their working memory capacity. Taken together, we found no evidence for a relationship between BMW-3 scores and cognitive abilities.^1^

Finally, we used a subsample of 230 participants from study 5 (see Table S5 in the supplement for descriptives and zero-order correlations) to assess the relationship between BMW-3 factors and self-reported attentional control. The model we specified provided a good account of the data, χ^2^(86) = 126.87, *p* = 0.003, CFI = 0.96, RMSEA = 0.05 (90% CI = [0.03; 0.06]). Participants with greater attentional control were less prone to unintentional mind wandering, *r* =  − 0.75, *p* < 0.001, 90% CI = [− 0.84; − 0.66], quicker to recognize when their mind had wandered, *r* = 0.39, *p* < 0.001, 90% CI = [0.27; 0.52], but were neither more nor less likely to let their thoughts drift intentionally, *r* =  − 0.05, *p* = 0.553, 90% CI = [− 0.19; 0.09], than those with lower attentional control.

#### Criterion validity

**Fig. 3:**
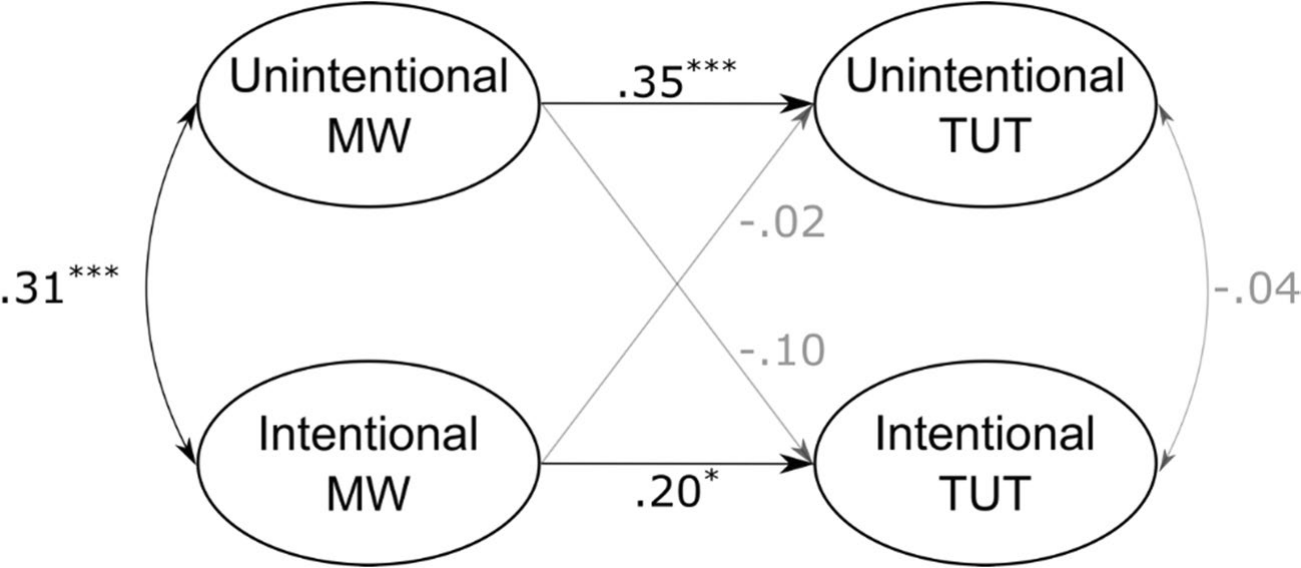
Results of the latent regression of unintentional and intentional task-unrelated thoughts on BMW-3 factors. MW = mind wandering; TUT = task-unrelated thoughts. *N* = 230. ^*^ *p* < .05. ^***^ *p* < .001

***Online thought-probing procedure.*** We evaluated the criterion validity of the BMW-3 by regressing latent factors of unintentional and intentional task-unrelated thoughts measured while participants completed three experimental tasks onto the latent factors of the three-factor model of BMW-3 items (see Fig. 3 and Table S6 in the supplementary material for descriptive statistics as well as factor loadings of all indicators). The structural equation model provided a good account of the data, χ^2^(184) = 270.63, *p* < 0.001, CFI = 0.94, RMSEA = 0.05 (90% CI = [0.03; 0.06]). Results of this analyses supported the criterion validity of the BMW-3, as participants more prone to unintentional mind wandering were also more prone to unintentionally think about something different than the current task in the lab, whereas participants letting their mind intentionally wander during routine activities were also more likely to intentionally think about something different than the current task in the lab. Fixing the two nonsignificant regression paths to zero slightly improved model fit, ∆AIC = 3.

In a follow-up analysis, we also explored whether participants’ meta-awareness of mind wandering predicted the occurrence of unintentional task-unrelated thoughts during experimental tasks. The adjusted model provided a good account of the data, χ^2^(266) = 369.23, *p* < 0.001, CFI = 0.94, RMSEA = 0.04 (90% CI = [0.03; 0.05]), but we found no evidence for any relationship between the meta-awareness of mind wandering and participants’ self-reported task-unrelated unintentional or intentional thoughts, all *r*s ≤|.10|, all *p*s ≥ 0.281.

#### Ecological momentary assessment of mind wandering.

We evaluated the criterion validity of the BMW-3 by predicting participants’ mind wandering in daily life assessed by ecological momentary assessment with the intentional and unintentional mind wandering scales with a generalized mixed model (see Table 5 for model results).^2^ Participants more prone to unintentional mind wandering reported higher rates of task-unrelated thoughts when probed throughout the day than did participants less prone to unintentional mind wandering, *OR* = 1.19, *p* = 0.031, 90% CI = [1.04; 1.35]. Participants’ disposition to let their thoughts drift intentionally, however, was not related to their everyday experience of task-unrelated thoughts, *OR* = 1.01, *p* = 0.924, 95% CI = [0.88; 1.15]. Hence, only unintentional, and not intentional, mind wandering assessed by the BMW-3 predicted participants’ everyday task-unrelated thoughts.

**Table 5:** Results of the generalized mixed model predicting everyday mind wandering

	Fixed effects			
	*OR*	*SE*	90% *CI*	*p*
Intercept	0.55	0.05	0.47–0.64	< .001
Within-person effects				
Time of day	0.94	0.02	0.90–0.98	.013
Covariates				
Unintentional mind wandering	1.19	0.09	1.04–1.35	0.031
Intentional mind wandering	1.01	0.08	0.88–1.15	0.924
	Variances of random effects			
σ^2^	3.29			
τ_00_ Participant	0.20			
τ_11_ Day (nested in participants)	0.01			
Marginal *R*^2^ / Conditional *R*^2^	0.01 / 0.07			

*N* = 88

In addition, we again conducted a follow-up analysis to explore whether participants’ meta-awareness of mind wandering predicted the occurrence of mind wandering in daily life by adding this scale as a predictor into the model and found no evidence for a relationship between the two, *OR* = 1.01, *p* = 0.912, 95% CI = [0.73; 1.48].

***Depressive symptoms.*** We further evaluated the criterion validity of the BMW-3 by regressing a latent depressive symptoms factor onto the latent factors of the three-factor model of BMW-3 items (for descriptives and zero-order correlations, see Table S7 in the supplementary material). The SEM provided a good account of the data, χ^2^(112) = 129.37, *p* = 0.125, CFI = 0.99, RMSEA = 0.03 (90% CI = [0.00; 0.05]). Results of these analyses supported the criterion validity of the BMW-3. Participants who were more prone to unintentional mind wandering were also more likely to experience depressive symptoms, β = 0.31, *p* = 0.002, 90% CI = [0.15; 0.47]. In comparison, the intentional mind wandering and the meta-awareness of mind wandering scales were not significantly related to depressive symptomatology, all βs ≤|.22|, all *p*s ≥ 0.086.

In addition, we tested whether the BMW-3 was able to distinguish between participants showing concerning levels of depressive symptoms (CES-D ≥ 22; *n* = 61) and participants showing low levels of depressive symptoms (CES-D < 22; *n* = 113). Participants with more unwanted episodes of mind wandering were more likely to be classified as showing concerning levels of depressive symptoms, *OR* = 2.09, *p* = 0.002, 90% CI = [1.43; 3.12], whereas the intentional mind wandering scale, *OR* = 1.25, *p* = 0.243, 90% CI = [0.92; 1.72], and the meta-awareness of mind wandering scale, *OR* = 0.72, *p* = 0.133, 90% CI = [0.49; 1.03], did not distinguish between the two groups.

***Emotion regulation.*** We further evaluated the criterion validity of the BMW-3 by regressing the three correlated latent factors of the emotion regulation strategies reappraisal, suppression, and rumination onto the latent factors of the three-factor model of BMW-3 items (for descriptives and zero-order correlations, see Table S8 in the supplementary material). The SEM provided an acceptable account of the data, χ^2^(310) = 548.27, *p* < 0.001, CFI = 0.85, RMSEA = 0.07 (90% CI = [0.06; 0.08]). Notably, the three subscales of the BMW-3 showed quite distinct relations to the three emotion regulation strategies (see Table 6). Participants who were more prone to unintentional mind wandering were more likely to regulate their emotions using suppression, while those more prone to intentional mind wandering were more likely to regulate their emotions using rumination. In addition, we found that individuals who were more aware of their thought states were more likely to use cognitive reappraisal as an emotion regulation strategy.

**Table 6:** Latent regression of emotion regulation strategies on BMW-3 scales

	Dependent variable					
	Cognitive reappraisal		Suppression		Rumination	
	β	90% CI	β	90% CI	β	90% CI
UI-MW	.00	-.18 – .19	.24^*^	.06 – .42	-.04	-.22 – .15
I-MW	.11	-.04 – .25	.13	-.01 – .28	.19^*^	.05 – .33
MA-MW	.32^**^	.14 – .50	-.03	-.22 – .16	-.01	-.20 – .17

UI-MW: unintentional mind wandering; I-MW: intentional mind wandering; MA-MW: meta-awareness of mind wandering; *N* = 174

^*^ *p* < .05. ^**^ *p* < .01

#### Validation of the English version of the BMW-3

Items of the English version of the scale showed mean values ranging from 1.98 to 3.01 and homogeneous standard deviations ranging from 1.06 to 1.38. Deviations of items and scales from normal distributions were negligible (see Figure S2 for the distributions of individual item scores and Table S8 for the correlation matrix); all skew values were ≤|1.33| and all kurtosis values were ≤|1.40|. Internal consistencies of all three scales were acceptable to good, with α = 0.72 for the UI-MW scale, α = 0.83 for the I-MW scale, and α = 0.79 for the MA-MW scale.

**Fig. 4:**
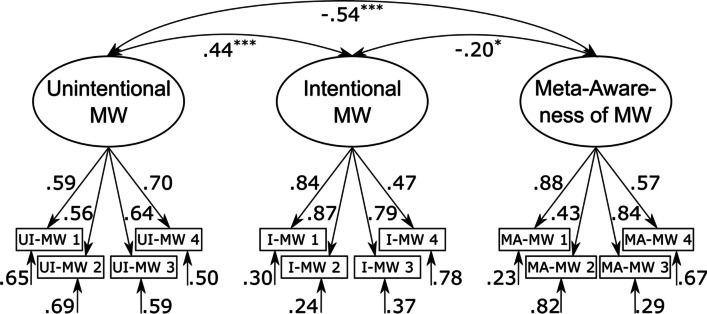
Graphical illustration of the best-fitting CFA model of the English version. Standardized factor loadings, correlation coefficients, and residual variances are shown next to paths. MW: mind wandering; UI-MW: unintentional mind wandering; I-MW: intentional mind wandering; MA-MW: meta-awareness of mind wandering. ^*^ *p* < .01. ^***^ *p* < .001*. N* = 215

**Factor structure.** The three-factor model consistent of a latent *unintentional mind wandering*, a latent *intentional mind wandering*, and a latent *meta-awareness* of mind wandering factor that were allowed to correlate freely with each other (see Fig. 4) provided an acceptable account of the data, χ^2^(51) = 128.82, *p* < 0.001, CFI = 0.92, RMSEA = 0.08 (90% CI = [0.07; 0.10]).

Model fit of all alternative models was worse, ∆AIC ≥ 60. In detail, the alternative model with three orthogonal factors, χ^2^(54) = 194.83, *p* < 0.001, CFI = 0.85, RMSEA = 0.11 (90% CI = [0.09; 0.13]), ΔAIC = 60, the alternative two-factor model with a latent *general mind wandering factor* and a latent *meta-awareness of mind wandering* factor, χ^2^(53) = 272.84, *p* < 0.001, CFI = 0.77, RMSEA = 0.14 (90% CI = [0.12; 0.16]), ΔAIC = 140, and the alternative one-factor model with a latent *general mind wandering factor*, χ^2^(54) = 531.11, *p* < 0.001, CFI = 0.49, RMSEA = 0.20 (90% CI = [0.19; 0.22]), ΔAIC = 396, all provided less adequate accounts of the data.

Subsequently, we evaluated the psychometric properties of the BMW-3 based on the best-fitting three-factor solution with correlated factors (see Fig. 4). Factor loadings were satisfactory (all βs ≥ 0.43). We observed a negative correlation between meta-awareness and unintentional mind wandering, *r* =  − 0.54, *p* < 0.001, 90% CI = [− 0.65; − 0.42], as well as intentional mind wandering, *r* =  − 0.20, *p* = 0.008, 90% CI = [− 0.33; − 0.08], which indicates that individuals who are more aware of their thought states are less likely to experience unwanted as well as intended episodes of mind wandering. Most importantly, intentional and unintentional mind wandering were only moderately correlated, *r* = 0.44, *p* < 0.001, 90% CI = [0.31; 0.56], which indicates that the questionnaire can successfully distinguish between the two types of mind wandering (although this correlation was numerically weaker in the German samples).

##### **Measurement invariance of the German and English versions**

We tested the German and English versions on three levels of measurement invariance: configural invariance (i.e., the same model holds for both versions), metric invariance (i.e., factor loadings are the same across both versions), and scalar invariance (i.e., intercepts and factor loadings are the same across both versions). Although absolute indices of model fit were consistent with scalar invariance (see Table 7), model comparisons favored metric over scalar invariance, suggesting that differences between German- and English-speaking participants should only be investigated with structural equation models accounting for differences in item intercepts between groups. In addition, the data were consistent with the assumption of equal covariances of latent variables across groups, ∆AIC = 0, indicating that the latent correlations between unintentional mind wandering, intentional mind wandering, and meta-awareness of mind wandering were equal across the two groups.

**Table 7:** Measurement invariance testing

	Level	χ²	*df*	*p*	CFI	RMSEA [90% CI]	AIC
DE vs. EN	Configural	296.67	102	<.001	.95	.06 [0.05; 0.07]	34444
	Metric	305.79	111	<.001	.95	.06 [0.05; 0.07]	34435
	Scalar	420.12	120	<.001	.93	.07 [0.06; 0.08]	34532
Lab vs. online	Configural	232.41	102	<.001	.96	.06 [0.05; 0.07]	27054
	Metric	239.48	111	<.001	.96	.05 [0.04; 0.06]	27043
	Scalar	255.84	120	<.001	.96	.05 [0.04; 0.06]	27041

*df* = degrees of freedom; CFI = comparative fit index; RMSEA = root mean square error of approximation; CI = confidence interval; AIC = Akaike information criterion. *N*_German_ = 823 (online = 277, lab = 546). *N*_English_ = 215.

Additionally, we ran analyses for measurement invariance between lab vs. online data collection in the German samples (see Table 7). For this comparison, we found evidence in favor of scalar invariance, indicating that the type of data collection did not affect the measurement of the three BMW-3 facets. Likewise, data were consistent with the assumption of equal covariances of latent variables across groups, ∆AIC = 2.

## Construct validity

***Measures of mind wandering and mindfulness.*** We assessed the convergent validity of the BMW-3 with related measures of mind wandering and mindfulness by computing correlations between the latent factors of the three-factor BMW-3 model and those of the MW-S, MW-D, and MAAS. The structural equation model provided an acceptable account of the data, χ^2^(238) = 486.56, *p* < 0.001, CFI = 0.90, RMSEA = 0.07 (90% CI = [0.06; 0.08]).

Correlations between the English versions of the BMW-3 and measures of mind wandering and mindfulness were largely comparable to the German version and supported the convergent and discriminant validity of the BMW-3 scales (see Table 8). As expected, and similar to the German sample results, we observed a high correlation between the UI-MW and MW-S scales, *r* = 0.72, *p* < 0.001, 90% CI = [0.63; 0.81], supporting the notion that both scales measure unintentional/spontaneous mind wandering. Moreover, we also observed a high correlation between the I-MW and MW-D scales, *r* = 0.73, *p* < 0.001, 90% CI = [0.66; 0.80], supporting the notion that both scales measure intentional/deliberate mind wandering. In addition, we observed a moderate correlation between the MA-MW scale and the MAAS, *r* = 0.58, *p* < 0.001, 90% CI = [0.49; 0.67], demonstrating that the two scales measure related but not identical facets of mindful awareness.

**Table 8:** Means, standard deviations, and latent correlations between the English versions of the BMW-3, the MW-S, MW-D, and MAAS

	*M*	*SD*	UI-MW	I-MW	MA-MW	MW-S	MW-D
UI-MW	2.62	0.83					
I-MW	2.25	1.07	.43^***^				
MA-MW	2.41	0.89	−.52^***^	−.20^*^			
MW-S	4.72	1.43	.72^***^	.42^***^	−.53^***^		
MW-D	4.64	1.59	.38^***^	.73^***^	−.14	.55^***^	
MAAS	3.44	0.90	−.47^***^	−.27^**^	.58^***^	−.56^***^	.29^***^

UI-MW: unintentional mind wandering; I-MW: intentional mind wandering; MA-MW: meta-awareness of mind wandering; MW-S: Mind Wandering: Spontaneous; MW-D: Mind Wandering: Deliberate; MAAS: Mindful Attention Awareness Scale. *M* and *SD* are used to represent the mean and standard deviation, respectively. *N *= 215.

^*^p < .05. ^**^p < .01. ^***^p < .001.

***Big Five personality traits.*** We evaluated the convergent and discriminant validity of the English version of the BMW-3 by regressing the latent factors of the three-factor model onto the correlated latent factors of Big Five personality traits measured by the IPIP questionnaire. The structural equation model provided a good account of the data, χ^2^(324) = 510.88, *p* < 0.001, CFI = 0.93, RMSEA = 0.05 (90% CI = [0.04; 0.06]).

The three BMW-3 factors showed overlapping associations with Big Five personality traits that were largely consistent with results from the German sample (see Table 9). Those who scored higher on conscientiousness and emotional stability were less prone to unintentional mind wandering, quicker to recognize it, and less likely to let their thoughts drift during routine activities. None of the other Big Five personality traits were related to the BMW-3. Most notably, we did not replicate the (modest but significant) relationship between openness to experience and intentional mind wandering that we observed in the German sample, given the regression coefficient of β = 0.02 not falling into the German sample’s 90% CI.

**Table 9:** Means and standard deviations of Big Five personality traits and results of the latent regression of BMW-3 factors on Big Five personality traits in the English sample

	*M*	*SD*	Dependent variable					
			UI-MW		I-MW		MA-MW	
			β	90% CI	β	90% CI	β	90% CI
O	3.60	0.64	.08	-.04 – .20	.02	-.10 – .14	.13	.01 – .25
C	3.37	0.67	-.38^***^	-.52 – -.25	-.34^***^	-.47 – -.22	.27^**^	.14 – .41
E	2.69	0.83	-.03	-.17 – .11	.03	-.10 – .17	-.09	-.22 – .05
A	3.80	0.64	.07	-.05 – .19	.06	-.07 – .18	.00	-.12 – .12
N	2.68	0.84	-.47^***^	-.59 – -.34	-.19^*^	-.32 – -.06	.22^**^	.09 – .35

UI-MW: unintentional mind wandering; I-MW: intentional mind wandering; MA-MW: meta-awareness of mind wandering; O: Openness to Experience; C: Conscientiousness; E: Extraversion; A: Agreeableness; N: Emotional Stability (i.e., higher values indicate higher emotional stability). *M* and *SD* are used to represent the mean and standard deviation, respectively. *N* = 215

^*^ *p* < .05. ^**^ *p* < .01. ^***^ *p* < .001

## Discussion

We validated the German and English versions of the BMW-3, a novel questionnaire that conceptualizes mind wandering as task-unrelated thought and reflects three dimensions of mind wandering, across student and general population samples. Results supported a three-factorial structure of self-reported mind wandering experiences (unintentional mind wandering, intentional mind wandering, and meta-awareness of mind wandering) and scalar measurement invariance of the German and English version. The BMW-3 showed good convergent validity with existing measures of mind wandering and mindfulness and was related to conscientiousness, emotional stability, and openness (in German samples) as well as self-reported attentional control. Moreover, it predicted the propensity to report in-the-moment mind wandering experiences inside and outside the lab, the frequency of experiencing depressive symptoms, and the use of functional and dysfunctional emotion regulation strategies. Our findings thus support the usefulness of the BMW-3 for basic and clinical research. In the remainder of this Discussion, we discuss the psychometric properties of the BMW-3, its validity, and implications for its use in different research contexts and consider limitations of the present validation studies.

### Psychometric properties

Confirmatory factor analyses unequivocally supported the theoretically proposed three-factor structure for both the German and the English versions of the questionnaire. The three distinct factors of unintentional mind wandering, intentional mind wandering, and meta-awareness of mind wandering demonstrated acceptable to good internal consistencies, ranging from 0.73 to 0.83, and moderate to high test–retest correlations, ranging from 0.41 to 0.73. Consequently, these three scales can be used to effectively assess individual differences in mind wandering in various research settings. In both language versions, factor loadings were high except for item I-MW 4 (“I actively use the time during routine tasks to mull over other things in the meanwhile”), which taps into a different aspect of intentional mind wandering than the other three items of the subscale. Unlike the other three items, which assess whether individuals let their minds wander to pass time, this item evaluates whether individuals use their spare mental time to ponder. As the item reflects an important facet of intentional mind wandering, prospective rumination, and its factor loading was still acceptable, we decided to retain it in the final version of the questionnaire instead of maximizing internal consistency at the cost of construct coverage (Clark & Watson, 2019; Clifton, 2020).

### Three factors of mind wandering

Both the confirmatory factor analyses and the validation results supported our choice to measure unintentional mind wandering, intentional mind wandering, and meta-awareness of mind wandering as three distinct factors. All three subscales showed good convergent validities. Whereas the unintentional and intentional mind wandering scales were highly correlated with existing measures of spontaneous and deliberate thought, respectively (*r*s > 0.70), the meta-awareness of mind wandering scale showed a substantial correlation with dispositional mindfulness (*r*s > 0.40). As the meta-awareness of mind wandering is a rather narrow construct in comparison to the broader construct of dispositional mindfulness, and considering that correlations tend to be underestimated if constructs are assessed at different levels of their respective hierarchy, the lower correlations of the meta-awareness subscale in comparison to the other two subscales should not cause any concerns, pending further validation evidence (Nesselroade & McArdle, 1997).

The validity of the three subscales was also supported by their relations to the Big Five personality traits. As expected, less conscientious participants were more likely to experience episodes of unintentional mind wandering and were also slower to realize when their thoughts had wandered off, as opposed to those who were less conscientious. In addition, we also found a negative association between conscientiousness and intentional mind wandering. These findings are consistent with previous research finding negative associations between conscientiousness and mind wandering (e.g., Caron et al., 2023; Müller et al., 2021; Robison et al., 2020). In addition, our findings align with prior studies showing that emotionally unstable individuals tend to worry a lot and have difficulties with controlling their thoughts. (Caron et al., 2023; Klein & Robinson, 2019; Widiger & Oltmanns, 2017). Specifically, we found that neuroticism predicted episodes of unintentional mind wandering and was negatively related to participants’ ability to recognize when their mind was wandering. Lastly, we found inconsistent evidence for a relationship between openness and intentional mind wandering. In the German-speaking sample, those with higher levels of trait openness were more inclined to intentionally let their minds wander during routine activities, as opposed to those with lower levels of trait openness. However, this correlation was not observed in the English-speaking sample. Taken together, these inconsistent findings add to the growing but inconclusive literature on the relationship between openness and intentional mind wandering and necessitate further research into potential moderators of this relationship (Ibaceta & Madrid, 2021; Kane et al., 2017; Robison et al., 2020; Rummel et al., 2022; Smeekens & Kane, 2016). Given the different correlations observed in the German- and English-speaking groups, cultural differences may be a potential moderator that should be more systematically investigated.

Lastly—and perhaps most impressively given possible concerns about the influence of shared-method variance—we found that the retrospective BMW-3 questionnaire successfully predicted the propensity for mind wandering both inside and outside the lab as measured by experience-sampling thought probes. Specifically, participants who were more susceptible to unintentional mind wandering also tended to unintentionally shift their thoughts away from the current task in the lab, while those who deliberately let their minds wander during routine activities were also more inclined to intentionally shift their thoughts away from the current task in the lab. These highly specific correlations of the two types of mind wandering support the necessity to distinguish between them (Carriere et al., 2013; Seli et al., 2016a, 2016b) and provide compelling evidence for the criterion validity of the unintentional and intentional mind wandering subscales of the BMW-3.

One limiting condition of our lab-based study is that it did not include a probe-based assessment of the meta-awareness of thought, as no agreed-upon measure exists in the field (Bernstein et al., 2019; Chu et al., 2023). When exploring whether the meta-awareness subscale of the BMW-3 predicted the occurrence of probe-caught task-unrelated thoughts, we did not find a significant relationship. This finding is not unexpected, as the meta-awareness subscale primarily measures the promptness with which an individual recognizes mind wandering, rather than their ability to refocus on the task at hand. A relationship between the meta-awareness scale and mind wandering rates would only be anticipated if individuals with higher meta-awareness were also more adept at re-engaging with ongoing tasks upon realizing their distraction. Nevertheless, the lack of any findings supporting the criterion validity of this subscale necessitates further research including measures that more heavily rely on one’s meta-awareness of mind wandering than probe-caught methods. A typical such measure is the self-caught method, where people are asked to press a key when they notice that their mind has wandered off. A recent review by Chu et al. (2023) recommended relating data on mind wandering with meta-awareness (i.e., using the self-caught method) to data on mind wandering without meta-awareness (i.e., using the probe-caught method) when conducting lab assessments to measure an individual's meta-awareness of mind wandering. Consequently, future studies could determine the ratio of self-caught to probe-caught mind wandering or the ratio of self-caught (aware) errors to unaware errors to gauge the level of meta-awareness in the lab and validate the meta-awareness subscale of the BMW-3.

Similar to other questionnaires of mind wandering such as the MW-S, MW-D, and MWQ, the BMW-3 was also able to predict participants’ mind wandering in daily life assessed with EMA methods. Consistent with previous research, unintentional, but not intentional, mind wandering assessed by the BMW-3 predicted participants’ rates of everyday task-unrelated thoughts (Ostojic-Aitkens et al., 2019). It remains an open question whether this is a methodological artifact that can be resolved by asking individuals about the intentionality of their task-unrelated thoughts in EMA probes. Alternatively, scales of intentional mind wandering may not be effective in predicting intentional task-unrelated thoughts in daily life because such thoughts occur infrequently (Rummel et al., 2022). Overall, these findings both inside and outside the lab support the criterion validity of the BMW-3 and also emphasize opportunities for further research.

### Relation between mind wandering and cognitive abilities

Cognitive accounts of mind wandering assume that people with better cognitive abilities are less prone to experience involuntary lapses of attention and are better able to regulate their mind wandering in accordance with current task demands than people with worse cognitive abilities (Robison et al., 2020; Rummel & Boywitt, 2014; Unsworth et al., 2021). We thus expected the unintentional mind wandering trait and mind wandering meta-awareness factors to be negatively associated with cognitive abilities as assessed with a broad test battery (BIS) and with two complex span tasks. However, we did not observe any such correlations. One reason for these null findings may be that cognitive abilities are more strongly associated with the frequency of task-unrelated thought phenomena in the laboratory than in everyday life (Kane et al., 2007), whereas the BMW-3 has been developed to assess task-unrelated thought occurrences in everyday life. Furthermore, in line with a multifaceted approach to mind wandering (Robison et al., 2020), one can assume that cognitive abilities are most strongly related to mind wandering in situations that are cognitively demanding, such as when carrying out demanding laboratory tasks or when attempting to focus during challenging everyday activities (Kane et al., 2007, 2017). However, we also found little evidence for a relationship between participants’ task-unrelated thought in the laboratory and their cognitive abilities in the present study. Our findings indicate that task demand is just one of many factors, including task interest, alertness level, sleepiness, physiological and mental state of being, and so on, that contribute to unintentional mind wandering in general, as measured by questionnaires. Indeed, some prior work has also found null associations between cognitive abilities and mind wandering as assessed with a questionnaire (Löffler et al., 2022). Nevertheless, it remains thought-provoking that correlations between cognitive abilities and all three BMW-3 factors were close to zero, even though the unintentional and intentional mind wandering factors were related to unintentional and intentional task-unrelated thought rates assessed during a cognitive laboratory task with high demands. Future research is necessary to resolve this puzzle.

### Clinical validity

Some evidence for the clinical utility of the BMW-3 stems from its relationship with depressive symptoms and the self-reported use of functional and dysfunctional emotion regulation strategies. As anticipated, the unintentional mind wandering subscale effectively differentiated between participants exhibiting elevated levels of depressive symptoms and those displaying minimal levels. This finding is consistent with the observation that individuals with depression are often consumed by thoughts that pertain to the past (Hamlat et al., 2015), which would manifest as a higher propensity to experience unwanted episodes of mind wandering (Chaieb et al., 2022).

Moreover, we found specific correlations between particular BMW-3 scales and the differential employment of the emotion regulation strategies reappraisal, suppression, and rumination. Those individuals with a greater tendency towards unintentional mind wandering were more inclined to regulate emotions through suppression, whereas those with a greater tendency towards intentional mind wandering were more likely to regulate emotions through rumination. In addition, individuals who were more aware of their thought states were more likely to use cognitive reappraisal as an emotion regulation strategy.

These findings support the hypothesis that people leverage mind wandering to regulate their negative emotions (Kruger et al., 2020). However, because the present study was correlational, strong conclusions regarding the causality between mind wandering and emotion regulation can only be drawn from further experimental research. Quite intriguingly, the present findings suggest that episodes of intentional mind wandering may be specifically used to reflect about one’s feelings and thoughts regarding a prior negative event through rumination. Prior research has indicated that rumination is closely associated with refocusing on planning as an emotion regulation strategy, and individuals have been found to engage in future planning during intentional mind wandering episodes (Feliu-Soler et al., 2017; Kvavilashvili & Rummel, 2020; Loch et al., 2011). Therefore, our results suggest that some people may intentionally engage in mind wandering to first reflect on a prior negative event and then make plans to manage or alter the situation. If rumination during intentional mind wandering primarily occurs in the context of future-oriented thinking, its classification as a primarily dysfunctional emotion regulation strategy should be reconsidered (Garnefski & Kraaij, 2007; Loch et al., 2011). However, because we cannot infer causality from correlational data, it is also possible that a reversed causality may exist. For instance, engaging in suppression might lead to episodes of unintentional mind wandering because people regulate only their expression of thoughts and feelings, not the thoughts and feelings themselves (Gross, 1998). This may cause them to continue thinking about the events evoking negative feelings. Additionally, the cognitive costs associated with suppression may make individuals who use this emotion regulation strategy more prone to distraction by unintentional task-unrelated thoughts (Richards & Gross, 1999). Furthermore, there may be bidirectional causal relationships between different emotion regulation strategies and various types of mind wandering. Therefore, future research should experimentally manipulate participants’ use of these emotion regulation strategies to determine the direction of the relationship.

Moving forward, it will also be important to examine the clinical validity of the BMW-3 and its subscales in clinical samples. Given that mind wandering has been implicated in various psychological disorders, such as clinical depression, OCD, and ADHD (Chaieb et al., 2022; Franklin et al., 2017; Seli et al., 2017), the BMW-3 could be used to identify individuals who may be at risk for developing these disorders or for monitoring treatment progress. However, more research is needed to establish the clinical validity of the BMW-3.

## Limitations

The current validation studies have limitations in terms of sample sizes and compositions. The test–retest study involved only 47 participants, so the test–retest correlations of the three scales must be considered as tentative evidence for their temporal stability. However, previous research has shown that the MW-S and MW-D scales also show a high test–retest correlation across two weeks and that probe-based measures of task-unrelated thoughts are consistent even over a six-month period (Marcusson-Clavertz & Kjell, 2019; Rummel et al., 2022), which supports the preliminary findings reported here. In the future, it would be beneficial to more systematically investigate the temporal stability and consistency of the BMW-3 scales using methods such as latent state–trait analysis (Steyer et al., 1992, 2015). Previous research on the trait and state characteristics of task-unrelated thoughts measured in the lab has shown that the propensity for mind wandering in the lab can be conceived of as a stable trait that is less affected by situational factors than typically assumed (Rummel et al., 2022). This is rather surprising, as changes in current motivational, affective, and physiological states have been shown to systematically induce changes in (mean) rates of mind wandering (Robison & Unsworth, 2018; Rummel & Nied, 2017; Smallwood et al., 2009). Because a controlled laboratory setting may reduce the effect of situational factors of mind wandering, it would be interesting to explore the trait and state characteristics of mind wandering in more naturalistic settings using the BMW-3.

A further extension of the BMW-3, not explored in the present study, is its adaptation to specific contexts such as academic settings, work environments, leisure activities, or creative tasks. Previous work has shown that, while being temporally stable, task-unrelated thoughts measured in the lab can be quite context-dependent when compared across different types of tasks (e.g., a reading and a working memory task; see Rummel et al., 2022; but see McVay & Kane, 2012). This suggests that mind wandering, as measured by the BMW-3 or other questionnaires, may also differ across various daily life contexts. For instance, individuals who do not typically let their minds wander during work-related activities might still experience mind wandering during creative tasks. Although systematically studying the impact of different everyday contexts was beyond the scope of this study, it represents a promising avenue for future research.

Another limitation is that we only validated the scales in Western European and North American samples. Consequently, the potential for cross-cultural generalization of our findings is limited. To assess the measurement invariance of the BMW-3 across more varied cultural contexts, it will be necessary to translate the scales into additional languages (but broader sampling simply across more German- and English-speaking populations will also be useful). Moreover, exploring other demographics, including adolescents with ADHD or individuals with clinical depression or OCD, may uncover additional areas where the BMW-3 may prove useful.

## Conclusion

On average, we spend a third or more of our waking hours engaging in self-generated thoughts that are unrelated to our ongoing activities (Kane et al., 2007; Killingsworth & Gilbert, 2010; Schooler et al., 2011). Here, we introduced a novel questionnaire, the BMW-3, that allows measuring individual differences in the frequency, nature, and awareness of these self-generated thoughts. The BMW-3 has been found to have good convergent validity when compared to existing measures of mind wandering and mindfulness, and is linked to traits such as conscientiousness, emotional stability, and openness, as well as self-reported attentional control. In addition, it has been shown to predict the likelihood of mind wandering inside and outside the lab, experiencing depressive symptoms, and the use of functional and dysfunctional emotion regulation strategies. Overall, these findings support the BMW-3’s utility for both basic and clinical research.

## Supplementary Information

Below is the link to the electronic supplementary material.

Supplementary file1 (DOCX 553 KB)

## Data Availability

The BMW-3 questionnaire and the data supporting the findings of the study are available in the Open Science Framework repository at https://osf.io/mxn3v/.

## Footnotes

1 We would like to thank an anonymous reviewer for suggesting to also explore the relationship between working memory capacity and unintentional as well as intentional task-unrelated thoughts measured with the online thought-probing procedure. For this, we used a subsample of the data (study 6), in which 230 participants also reported their thought states during three experimental tasks. The model showed an acceptable model fit, χ^2^(118) = 191.21, *p* < .001, CFI = 0.92, RMSEA = 0.05 (90% CI = [0.04; 0.07]). Similar to the findings reported for the BMW-3 above, we again found no significant association between participants’ working memory capacity and their inclination to report either unintentional task-unrelated thoughts, *r*_UN,WMC_ = .10, *p* = .364, 90% CI = [− .08; .27], or intentional task-unrelated thoughts, *r*_IN,WMC_ = .02, *p* = .857, 90% CI = [− .15; .19].

2 Not including time of day as a predictor did not change any of the main results, but slightly impaired overall model fit, χ^2^ = 6.17, *p* = .013. Participants’ disposition to let their mind wander unintentionally still predicted their everyday experiences of task-unrelated thoughts, *OR* = 1.19, *p* = .030, 90% CI = [1.04; 1.35], whereas their disposition to let their mind wander intentionally did not, *OR* = 1.01, *p* = .923, 90% CI = [0.88; 1.15].

## Declarations

### Open practices statement

The BMW-3 questionnaire and the data supporting the findings of the study are available in the Open Science Framework repository at https://osf.io/mxn3v/. The study materials will be shared upon request with the exception of the Berlin Intelligence Structure test, which is commercially licensed. The statistical analysis code needed to reproduce the analyses in the manuscript are available in the Open Science Framework repository at https://osf.io/mxn3v/. None of the experiments were preregistered.
