# Supplementary material for: The brief mind wandering three-factor scale (BMW-3)
Source: Behav Res Methods. 2024 Sep 11;56(8):8720–44. doi: 10.3758/s13428-024-02500-6 (PMC11525255; doi:10.3758/s13428-024-02500-6)
Supplement: Supplementary file 1 — Supplementary file1 (DOCX 553 KB) [file 13428_2024_2500_MOESM1_ESM.docx]

**Supplementary Material**

**Figure S1**

*Distributions of individual item scores in the German-speaking sample
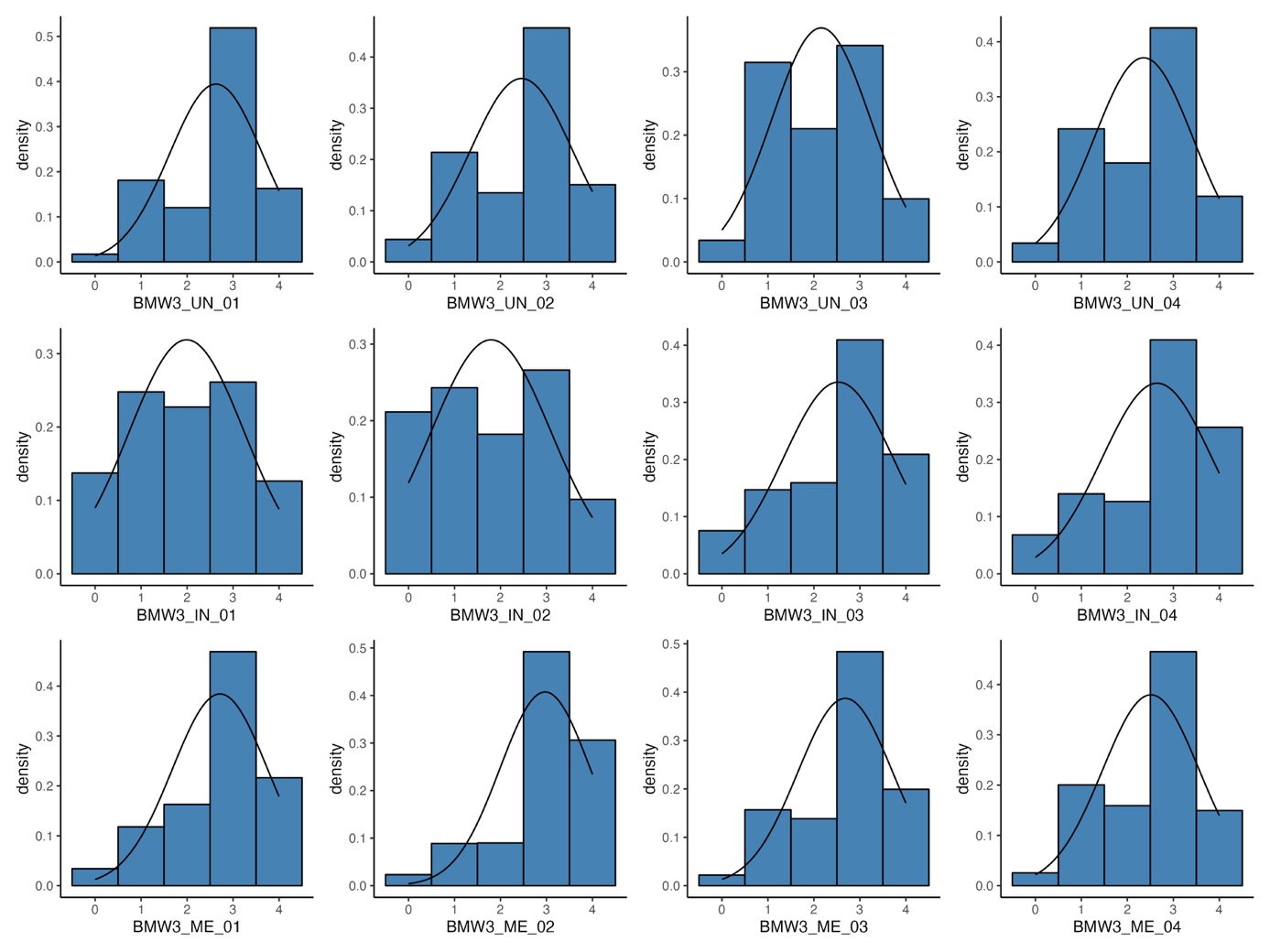
*

*Note.* BMW-3: Brief Mind Wandering Three-Factor Scale; BMW3_UN: Unintentional Mind Wandering scale of the BMW-3; BMW3_IN: Intentional Mind Wandering scale of the BMW-3; BMW_ME: Meta-Awareness of Mind Wandering scale of the BMW-3. *N* = 823

**Figure S2**

*Distributions of individual item scores in the English-speaking sample*
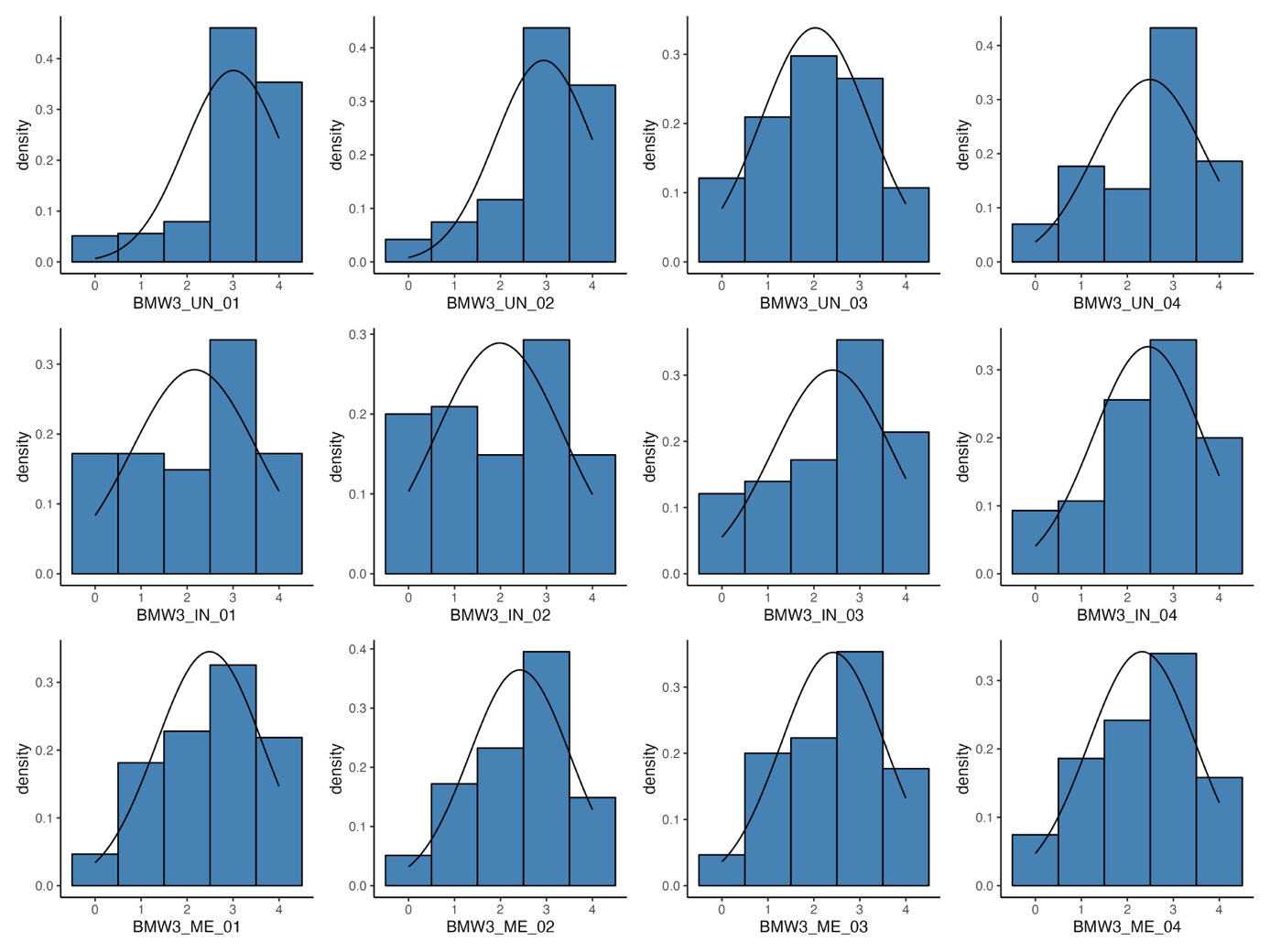


*Note.* BMW-3: Brief Mind Wandering Three-Factor Scale; BMW3_UN: Unintentional Mind Wandering scale of the BMW-3; BMW3_IN: Intentional Mind Wandering scale of the BMW-3; BMW_ME: Meta-Awareness of Mind Wandering scale of the BMW-3. *N* = 215.

**Table S1**

*Original German items*

|  | Item |
| --- | --- |
|  |  |
| UI-MW 1 | Während ich einen Vortrag höre, wandern meine Gedanken ohne mein Zutun zu anderen Dingen. |
| UI-MW 2 | Wenn ich Fernsehen schaue, gehen mir dabei ungewollt andere Dinge durch den Kopf |
| UI-MW 3 | Ich bin oft nicht voll bei der Sache. |
| UI-MW4 | Wenn ich einer Tätigkeit nachgehe, schweifen meine Gedanken wie von selbst zu anderen Dingen. |
| I-MW 1 | Ich lasse meine Gedanken treiben, damit die Zeit schneller vergeht |
| I-MW 2 | Ich schweife gezielt gedanklich ab, um dem Alltagstrott zu entfliehen |
| I-MW 3 | Ich lenke mich in eintönigen Situationen ab, indem ich meinen Gedanken freien Lauf lasse. |
| I-MW 4 | Ich nutze Routinetätigkeiten, um mir währenddessen Gedanken über andere Dinge zu machen. |
| MA-MW 1^1^ | Es dauert sehr lange, bis ich merke, dass meine Gedanken abgeschweift sind. |
| MA-MW 2 | Mir fällt schnell auf, wenn ich jemandem nicht aufmerksam zuhöre. |
| MA-MW 3^1^ | Es dauert eine Weile, bis mir auffällt, dass ich gedanklich nicht mehr bei der Sache bin. |
| MA-MW 4 | Ich bemerke sofort, wenn meine Gedanken nicht im Hier und Jetzt sind. |

**Table S2**

*Means, standard deviations, and correlations of the BMW-3 items in the German samples.*

| Item | *M* | *SD* | 1 | 2 | 3 | 4 | 5 | 6 | 7 | 8 | 9 | 10 | 11 |
| --- | --- | --- | --- | --- | --- | --- | --- | --- | --- | --- | --- | --- | --- |
| 1. UN_01 | 2.63 | 1.01 |  |  |  |  |  |  |  |  |  |  |  |
| 2. UN_02 | 2.46 | 1.11 | .40** |  |  |  |  |  |  |  |  |  |  |
| 3. UN_03 | 2.16 | 1.08 | .50** | .42** |  |  |  |  |  |  |  |  |  |
| 4. UN_04 | 2.35 | 1.08 | .49** | .38** | .63** |  |  |  |  |  |  |  |  |
| 5. ME_01 | 2.72 | 1.04 | -.28** | -.22** | -.34** | -.33** |  |  |  |  |  |  |  |
| 6. ME_02 | 2.97 | 0.98 | -.19** | -.07* | -.21** | -.10** | .36** |  |  |  |  |  |  |
| 7. ME_03 | 2.68 | 1.03 | -.28** | -.20** | -.31** | -.27** | .68** | .44** |  |  |  |  |  |
| 8. ME_04 | 2.51 | 1.05 | -.19** | -.10** | -.19** | -.23** | .56** | .39** | .58** |  |  |  |  |
| 9. IN_01 | 1.99 | 1.25 | .23** | .13** | .15** | .18** | -.09** | -.09** | -.08* | -.03 |  |  |  |
| 10. IN_02 | 1.79 | 1.31 | .14** | .16** | .15** | .15** | -.08* | -.04 | -.06 | -.04 | .60** |  |  |
| 11. IN_03 | 2.53 | 1.19 | .13** | .07 | .10** | .13** | -.03 | -.00 | .01 | -.02 | .53** | .57** |  |
| 12. IN_04 | 2.65 | 1.20 | .01 | .06 | .03 | .08* | .02 | .12** | .07 | .03 | .19** | .20** | .34** |

*Note.* *M* and *SD* are used to represent mean and standard deviation, respectively. * indicates *p* < .05. ** indicates *p* < .01. UN = unintentional mind wandering; ME = meta-awareness of mind wandering; IN = intentional mind wandering.

**Table S3**

*Means, standard deviations, and correlations of BMW3 average scale scores and z-standardized component scores of the BIS test.*

|  | Variable | *M* | *SD* | 1 | 2 | 3 | 4 | 5 | 6 |
| --- | --- | --- | --- | --- | --- | --- | --- | --- | --- |
| BMW3 | 1. UN | 2.39 | 0.76 |  |  |  |  |  |  |
|  | 2. IN | 2.25 | 0.90 | .21** |  |  |  |  |  |
|  | 3. ME | 2.68 | 0.81 | -.26** | .03 |  |  |  |  |
| Cog. Abilities | 4. zPC | 613.71 | 44.10 | .04 | .12 | -.04 |  |  |  |
|  | 5. zPS | 303.45 | 23.40 | .06 | .06 | .00 | .49** |  |  |
|  | 6. zM | 295.27 | 22.87 | -.02 | .01 | -.08 | .45** | .36** |  |
|  | 7. zC | 295.81 | 20.51 | -.07 | -.00 | .07 | .36** | .29** | .10 |

*Note.* *M* and *SD* are used to represent mean and standard deviation, respectively. * indicates *p* < .05. ** indicates *p* < .01. UN = unintentional mind wandering; ME = meta-awareness of mind wandering; IN = intentional mind wandering; PC = processing capacity; PS = processing speed; M = memory; C = creativity.

**Table S4**

*Means, standard deviations, and correlations of BMW3 average scores and WMC indicators.*

| Variable | *M* | *SD* | 1 | 2 | 3 | 4 | 5 | 6 |
| --- | --- | --- | --- | --- | --- | --- | --- | --- |
| 1. BMW3 UN | 2.38 | 0.79 |  |  |  |  |  |  |
| 2. BMW3 IN | 2.33 | 0.94 | .23** |  |  |  |  |  |
| 3. BMW3 ME | 2.74 | 0.83 | -.27** | -.06 |  |  |  |  |
| 4. SSpan B1 | 10.19 | 2.61 | -.04 | -.06 | .01 |  |  |  |
| 5. SSpan B2 | 10.14 | 2.90 | -.12 | -.04 | .06 | .46** |  |  |
| 6. Ospan B1 | 19.81 | 4.27 | .03 | -.02 | -.01 | .20** | .28** |  |
| 7. Ospan B2 | 20.35 | 3.98 | -.03 | -.04 | -.06 | .13* | .24** | .56** |

*Note.* *M* and *SD* are used to represent mean and standard deviation, respectively. * indicates *p* < .05. ** indicates *p* < .01. UN = unintentional mind wandering; ME = meta-awareness of mind wandering; IN = intentional mind wandering; SSpan = Spatial Span task; OSpan = Operation Span task; B = block.

**Table S5**

*Means, standard deviations, and correlations of BMW3 average scores and AC indicators.*

| Variable | *M* | *SD* | 1 | 2 | 3 | 4 |
| --- | --- | --- | --- | --- | --- | --- |
| 1. BMW3 UN | 2.34 | 0.82 |  |  |  |  |
| 2. BMW3 IN | 2.36 | 0.95 | .22** |  |  |  |
| 3. BMW3 ME | 2.81 | 0.79 | -.30** | -.12 |  |  |
| 4. ACS Focusing | 2.48 | 0.45 | -.50** | -.02 | .20** |  |
| 5. ACS Shifting | 2.71 | 0.40 | -.42** | -.00 | .36** | .59** |

*Note.* *M* and *SD* are used to represent mean and standard deviation, respectively. * indicates *p* < .05. ** indicates *p* < .01. UN = unintentional mind wandering; ME = meta-awareness of mind wandering; IN = intentional mind wandering; ACS = Attentional Control Scale.

**Table S6**

Descriptive statistics of the standardized indicators of BMW3 scales and online TUT rates and factor loadings for the SEM reported in Figure 2.

|  | Variable | | | | *M* | *SD* | 1 | 2 | 3 | 4 | 5 | 6 | 7 | 8 | 9 | 10 | 11 | 12 | 13 |
| --- | --- | --- | --- | --- | --- | --- | --- | --- | --- | --- | --- | --- | --- | --- | --- | --- | --- | --- | --- |
| 1 | BMW3 |  | UN |  | 2.34 | 1 |  |  |  |  |  |  |  |  |  |  |  |  |  |
| 2 |  |  | IN |  | 2.36 | 0.95 | .22** |  |  |  |  |  |  |  |  |  |  |  |  |
| 3 | TUT rate | UN | NB | easy | 1.73 | 1.09 | .07 | -.03 |  |  |  |  |  |  |  |  |  |  |  |
| 4 |  |  |  | difficult | 0.93 | 1.04 | .00 | -.05 | .21** |  |  |  |  |  |  |  |  |  |  |
| 5 |  |  | CMT | easy | 1.62 | 1.10 | .13 | -.05 | .29** | .13 |  |  |  |  |  |  |  |  |  |
| 6 |  |  |  | difficult | 1.34 | 1.11 | .17* | -.02 | .31** | .24** | .18* |  |  |  |  |  |  |  |  |
| 7 |  |  | MS | easy | 1.64 | 1.15 | .18* | -.03 | .27** | .12 | .42** | .36** |  |  |  |  |  |  |  |
| 8 |  |  |  | difficult | 1.61 | 1.22 | .04 | -.10 | .30** | .24** | .38** | .45** | .38** |  |  |  |  |  |  |
| 9 |  | IN | NB | easy | 0.65 | 0.89 | .06 | .23** | -.01 | .15* | -.02 | .10 | .06 | -.04 |  |  |  |  |  |
| 10 |  |  |  | difficult | 0.22 | 0.60 | .06 | .05 | .10 | .19** | -.03 | .11 | .10 | .04 | .31** |  |  |  |  |
| 11 |  |  | CMT | easy | 0.58 | 0.86 | .04 | .09 | .07 | .13 | .04 | .29** | .02 | .02 | .48** | .21** |  |  |  |
| 12 |  |  |  | difficult | 0.31 | 0.66 | .02 | .13 | .05 | .14* | -.06 | .16* | .04 | -.03 | .23** | .29** | .37** |  |  |
| 13 |  |  | MS | easy | 0.75 | 0.96 | .03 | .21** | -.02 | .05 | .10 | .13 | .11 | .14 | .37** | .09 | .38** | .24** |  |
| 14 |  |  |  | difficult | 0.46 | 0.82 | .04 | .16* | .09 | .09 | .00 | .14 | -.02 | .05 | .38** | .25** | .44** | .35** | .42** |

Note. *M* and *SD* are used to represent mean and standard deviation, respectively. * indicates *p* < .05. ** indicates *p* < .01. UN = unintentional mind wandering; ME = meta-awareness of mind wandering; IN = intentional mind wandering; TUT = task-unrelated thoughts, NB = N-Back task, CMT = Color-matching task, MS = Memory-scanning task.

**Table S7**

*Means, standard deviations, and correlations of BMW3 subscales with depressive symptoms and emotion regulation strategies.*

|  | *M* | *SD* | 1 | 2 | 3 | 4 | 5 | 6 | 7 |
| --- | --- | --- | --- | --- | --- | --- | --- | --- | --- |
| 1. BMW3 UN | 2.45 | 0.91 |  |  |  |  |  |  |  |
| 2. BMW3 IN | 2.15 | 0.94 | .13 |  |  |  |  |  |  |
| 3. BMW3 ME | 2.62 | 0.84 | -.43** | .01 |  |  |  |  |  |
| 4. Depression | 18.25 | 12.27 | .37** | .10 | -.32** |  |  |  |  |
| 5. Rumination | 3.53 | 0.73 | .09 | .17* | -.06 | .21** |  |  |  |
| 6. Reappraisal | 4.60 | 1.00 | -.16* | .20** | .27** | -.36** | .08 |  |  |
| 7. Suppression | 3.59 | 1.25 | .21** | .09 | -.13 | .34** | -.04 | -.03 |  |
| 8. Mood Regulation | 2.81 | 0.59 | -.24** | .04 | .34** | -.67** | -.19* | .46** | -.41** |

*Note.* *M* and *SD* are used to represent mean and standard deviation, respectively. * indicates *p* < .05. ** indicates *p* < .01. UN = unintentional mind wandering; ME = meta-awareness of mind wandering; IN = intentional mind wandering.

**Table S8**

*Means, standard deviations, and correlations of the BMW-3 items in the English sample*

| Variable | *M* | *SD* | 1 | 2 | 3 | 4 | 5 | 6 | 7 | 8 | 9 | 10 | 11 |
| --- | --- | --- | --- | --- | --- | --- | --- | --- | --- | --- | --- | --- | --- |
| 1. UN_01 | 3.01 | 1.06 |  |  |  |  |  |  |  |  |  |  |  |
| 2. UN_02 | 2.94 | 1.06 | .43** |  |  |  |  |  |  |  |  |  |  |
| 3. UN_03 | 2.03 | 1.18 | .37** | .30** |  |  |  |  |  |  |  |  |  |
| 4. UN_04 | 2.49 | 1.18 | .43** | .41** | .41** |  |  |  |  |  |  |  |  |
| 5. ME_01 | 2.49 | 1.16 | -.20** | -.19** | -.49** | -.37** |  |  |  |  |  |  |  |
| 6. ME_02 | 2.42 | 1.09 | -.02 | .07 | -.14* | -.01 | .34** |  |  |  |  |  |  |
| 7. ME_03 | 2.41 | 1.13 | -.24** | -.21** | -.43** | -.28** | .74** | .37** |  |  |  |  |  |
| 8. ME_04 | 2.32 | 1.17 | -.06 | -.01 | -.20** | -.10 | .50** | .47** | .47** |  |  |  |  |
| 9. IN_01 | 2.16 | 1.37 | .10 | .17* | .23** | .25** | -.18** | .04 | -.15* | .03 |  |  |  |
| 10. IN_02 | 1.98 | 1.38 | .09 | .17* | .26** | .33** | -.14* | .06 | -.13 | .04 | .75** |  |  |
| 11. IN_03 | 2.40 | 1.30 | .20** | .25** | .19** | .33** | -.20** | .05 | -.23** | -.05 | .65** | .68** |  |
| 12. IN_04 | 2.45 | 1.19 | .26** | .27** | .21** | .30** | -.13 | .12 | -.16* | .03 | .35** | .38** | .44** |

*Note.* *M* and *SD* are used to represent mean and standard deviation, respectively.* indicates *p* < .05. ** indicates *p* < .01. UN = unintentional mind wandering; ME = meta-awareness of mind wandering; IN = intentional mind wandering.
